# Comprehensive analysis of SQOR involvement in ferroptosis resistance of pancreatic ductal adenocarcinoma in hypoxic environments

**DOI:** 10.3389/fimmu.2025.1513589

**Published:** 2025-05-01

**Authors:** Quan Lin, Shiwei Guan, Minghui Peng, Kailun Zhang, Hewei Zhang, Taoming Mo, Haibo Yu

**Affiliations:** ^1^ Department of Hepatobiliary Surgery, Wenzhou Central Hospital, The Dingli Clinical Institute of Wenzhou Medical University, Wenzhou, Zhejiang, China; ^2^ Department of Pathology, Medical School of Nantong University, Nantong, Jiangsu, China

**Keywords:** pancreatic ductal adenocarcinoma, hypoxia, SQOR, ferroptosis, pathomics, deep learning

## Abstract

**Introduction:**

Pancreatic ductal adenocarcinoma (PDAC) exhibits higher hypoxia level than most solid tumors, and the presence of intratumoral hypoxia is associated with a poor prognosis. However, the identification of hypoxia levels based on pathological images, and the mechanisms regulating ferroptosis resistance, remain to be elucidated. The objective of this study was to construct a deep learning model to evaluate the hypoxia characteristics of PDAC and to explore the role of Sulfide quinone oxidoreductase (SQOR) in hypoxia-mediated ferroptosis resistance.

**Methods:**

Multi-omics data were integrated to analyze the correlation between hypoxia score of PDAC, SQOR expression and prognosis, and ferroptosis resistance level. A deep learning model of Whole Slide Images (WSIs) were constructed to predict the hypoxia level of patients. *In vitro* hypoxia cell models, SQOR knockdown experiments and nude mouse xenograft models were used to verify the regulatory function of SQOR on ferroptosis.

**Results:**

PDAC exhibited significantly higher hypoxia levels than normal tissues, correlating with reduced overall survival in patients. In slide level, our deep learning model can effectively identify PDAC hypoxia levels with good performance. SQOR was upregulated in tumor tissues and positively associated with both hypoxia score and ferroptosis resistance. SQOR promotes the malignant progression of PDAC in hypoxic environment by enhancing the resistance of tumor cells to ferroptosis. SQOR knockdown resulted in decreased cell viability, decreased migration ability and increased MDA level under hypoxic Ersatin induced conditions. Furthermore, SQOR inhibitor in combination with ferroptosis inducer has the potential to inhibit tumor growth *in vivo* in a synergistic manner.

**Discussion:**

This study has established a hypoxia detection model of PDAC based on WSIs, providing a new tool for clinical evaluation. The study revealed a new mechanism of SQOR mediating ferroptosis resistance under hypoxia and provided a basis for targeted therapy.

## 1 Introduction

Pancreatic ductal adenocarcinoma (PDAC), an aggressive malignancy originating from exocrine ductal cells, accounts for over 90% of pancreatic cancers and ranks as the third leading cause of cancer-related deaths in the United States, with a dismal 5-year survival rate of 11% (1–3). However, only 15-20% of patients with PDAC can undergo surgical resection due to its lack of specific clinical presentation in the early stages, early local invasion, and high metastatic potential (1, 4). Over 80% of patients present with unresectable disease at diagnosis, primarily due to vascular invasion or distant metastases, and are thus limited to palliative care (5). Patients with locally advanced or metastatic PDAC are typically deemed incurable and are limited to receiving palliative care. Despite the emergence of various therapies in recent years, including immune checkpoint inhibitors, the results in PDAC have been disappointing. There are still very few long-term survivors of PDAC. Therefore, new therapeutic strategies are urgently needed to improve patient prognosis.

Hypoxia occurs when intracellular oxygen levels decrease (6). Studies have shown that hypoxia promotes malignant behavior in cancer cells, including proliferation, migration, invasion, and increased resistance to immunotherapy, radiotherapy, and chemotherapy (7). Furthermore, a hypoxic environment alters the expression levels of genes that regulate metabolism and other processes. Hypoxia is the hallmark feature of PDAC, resulting from the disturbed tumor vasculature and dense fibrous stroma. The degree of hypoxia in PDAC is significantly higher than that in most solid tumors and is associated with poor prognosis of patients with PDAC. With the deepening understanding of the hypoxic microenvironment of PDAC, hypoxia has gradually become a key driver of PDAC and is regarded as a potential therapeutic target.

In recent years, with the development of artificial intelligence (AI) technologies, advances in deep learning in computational pathology have enabled Whole Slide Images (WSIs) to be used for automated cancer diagnosis and quantification of morphological phenotypes in the tumor microenvironment (TME) (8). While the use of WSIs for specific biologically meaningful studies is still rare and difficult to interpret due to the fact that deep models are referred to as black-box models, attempts to interpret the meaning can be of great help in biological studies. Among them, weakly supervised deep learning based on multi-instance learning (MIL) provides greater help in reducing pathologist annotations and improving image training at high resolution. Currently, the identification of hypoxia in PDAC tissues is mainly determined through laboratory tests or some hypoxic signs (e.g., lack of vascular manifestations) on imaging (9, 10). And there has not been any study on directly detecting through pathological H&E staining using deep learning methods.

While AI-driven pathomics provides tools to decode hypoxia-related features, the molecular mechanisms linking hypoxia to PDAC progression remain underexplored. Sulfide quinone oxidoreductase (SQOR), also known as SQRDL or SQR, located in mitochondria, is a membrane-bound flavoprotein of the glutathione reductase family and a key enzyme in the oxidative detoxification of sulfides (11). It can use ubiquinone as an electron acceptor to catalyze the two-electron oxidation of H2S to produce sulfur and transfer electrons from H2S to ubiquinone (12, 13). It has been shown that persulfide produced by SQOR-mediated sulfide oxidation may be an electron acceptor for the electron transfer chain, promoting mitochondrial ATP production (14). Increased expression of SQOR in mitochondria increased tolerance to hypoxia not only in the brain but also in the heart and liver (11). Recent studies have revealed that SQOR catalyzes the reduction of ubiquinone to ubiquinol via hydrogen selenide, a metabolic intermediate of selenium, thereby suppressing lipid peroxidation and ferroptosis (15).

Ferroptosis is a form of iron-dependent cell death driven by excessive lipid peroxidation and is associated with the development of various types of tumors and response to treatment (16, 17). Studies have shown that RAS-mutated cancer cells are sensitive to ferroptosis induction and that chemotherapeutic agents and ferroptosis inducers have synergistic effects in tumor therapy (18–20). KRAS, a member of the RAS GTPase family, is mutationally activated in over 90% of PDAC cases (21). In addition, a study in PDAC found that the combination of ferroptosis inducers and apoptosis inducers significantly increased the cytotoxicity of gemcitabine (22). There is now growing evidence of a strong correlation between hypoxia and ferroptosis. One study observed that the hypoxic TME promotes resistance to ferroptosis in solid tumors in a hypoxia-inducible factor 1α -dependent manner (23). In addition to HIF, increased activity of Nrf2, a major regulator of the antioxidant system, during hypoxia promotes HO-1 expression, thereby preventing ferroptosis (24). Therefore, the link and drivers between hypoxia and ferroptosis resistance deserve further exploration.

Through bioinformatics analysis, deep learning-based pathomics analysis, and *in vitro* experiments, this study aims to characterize hypoxic PDAC in the ecosystem and elucidate the correlation between SQOR and ferroptosis resistance under hypoxia, providing new therapeutic directions to improve the prognosis of PDAC patients with high hypoxia levels.

## 2 Methods

### 2.1 Data access

**Figure 1:**
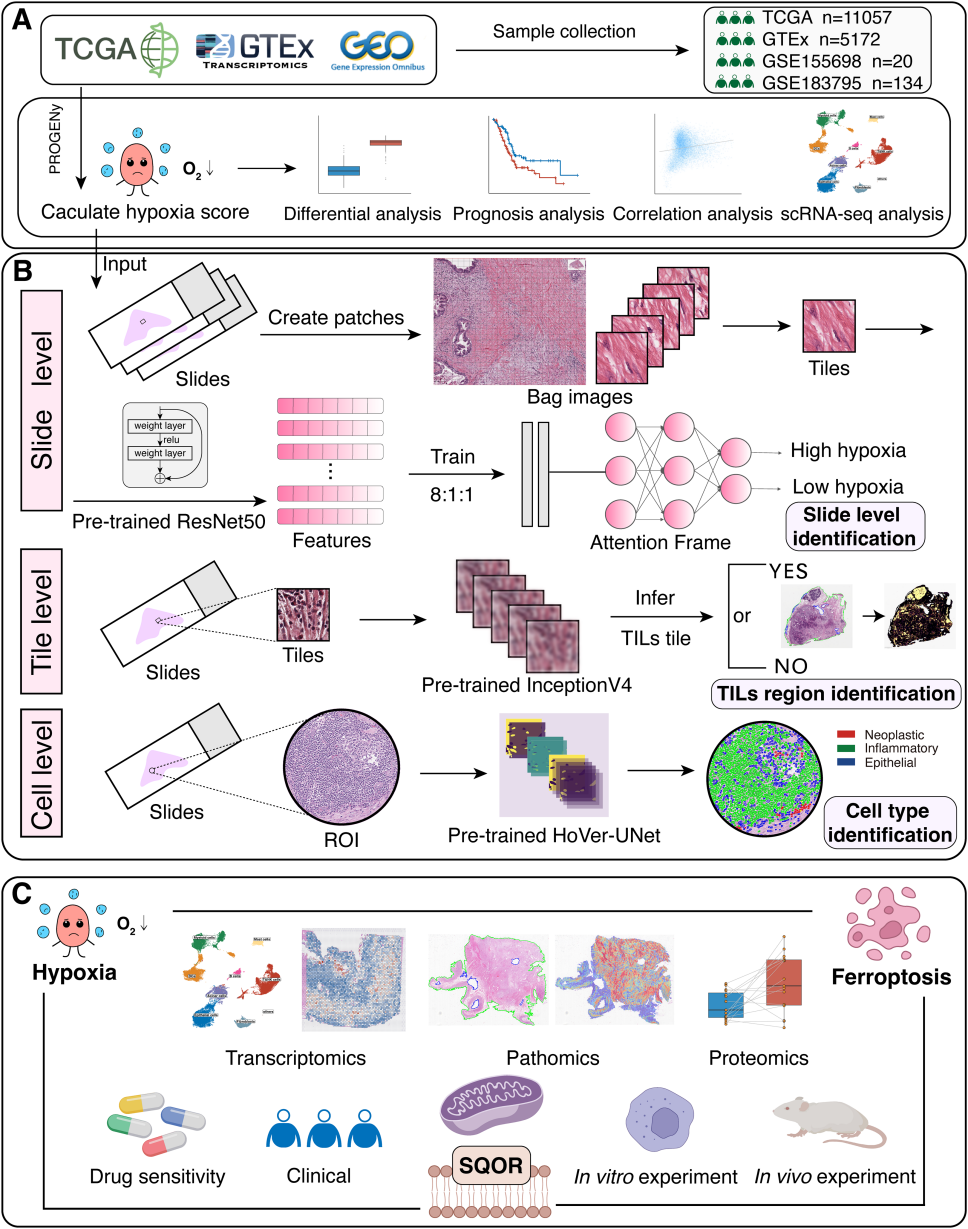
The workflow of our study. **(A)** TCGA, GTEx and GEO datasets were downloaded for bioinformatics analysis. Hypoxia scores were calculated using the “progeny” package. **(B)** Construct a MIL-based hypoxia discrimination model and interpret it at slide level, tile level and cell level. **(C)** The relationship between hypoxia, SQOR and ferroptosis was investigated by transcriptomics, pathomics, proteomics, drug sensitivity, clinical data analysis and *in vitro* experiments. TCGA, The Cancer Genome Atlas; GTEx, Genotype-Tissue Expression; scRNA-seq, single-cell RNA-seq; MIL, multi-instance learning; TILs, tumor-infiltrating lymphocytes; ROI, region-of-interest.

RNA sequencing (RNA-seq) data for tumors and normal tissues were obtained from the University of Cingifornia Sisha Cruz (UCSC) Xena database (https://xenabrowser.net/datapages/). We used The Cancer Genome Atlas (TCGA) data and Genotype-Tissue Expression (GTEx) data. Survival data for TCGA patients were downloaded from “PanCanAtlas Publications” (https://gdc.cancer.gov/about-data/publications/pancanatlas). The microarray dataset GSE183795, single-cell RNA-seq (scRNA-seq) dataset GSE155698 and spatial transcriptome (ST) dataset GSE235315 for pancreatic cancer were obtained from Gene Expression Omnibus (GEO) database (https://www.ncbi.nlm.nih.gov/geo/, **Figure 1A**, **Supplementary Table S1**). Proteomics data were obtained from Savage et al., and the data consists of epithelial-enriched cores, stroma-enriched cores and bulk tissue from tumor and normal tissues (25).

The clinical data and case specimens of this study were collected from 24 patients with PDAC confirmed by pathology in Wenzhou Central Hospital from January 2017 to December 2022, all of the above were diagnosed through pathology. This study was approved by the Ethics Committee of Wenzhou Central Hospital (approval number: 202402052106000086527), and the general clinical data of the patients were recorded (**Supplementary Table S2**). TNM staging was based on the TNM staging criteria for pancreatic cancer jointly developed by the American Joint Committee on Cancer (AJCC) and the International Union Against Cancer (UICC), both for patients with stage I-III.

### 2.2 Bulk RNA-seq data processing

Raw HTSeq-counts data obtained from the UCSC Xena database were utilized for normalization in this study. Initially, the effective gene lengths were calculated using the GENCODE v36 genome annotation file. Subsequently, the raw counts were transformed into Transcripts Per Million (TPM) values through a standardized method. To enhance the data distribution, the results were further converted to log2(TPM + 0.001). The conversion of Ensembl IDs to gene symbols was performed using the GENCODE v36 gene probe annotation file. The expression profile of the SQOR gene was extracted for subsequent analyses.

RNA-seq data from the GTEx and TCGA databases were integrated to assess hypoxia pathway activity and conduct differential expression analysis of SQOR across pan-cancer samples. The data processing workflow included the following steps: TPM data for GTEx normal tissues and HTSeq-counts raw data for TCGA tumor samples were retrieved from the UCSC Xena platform. Non-disease-related tissues were excluded, and tumor samples in TCGA were screened based on predefined criteria. The raw counts of TCGA samples were normalized into TPM values following the aforementioned procedure. Finally, the GTEx and TCGA expression matrices were merged to form an integrated dataset, which was stratified by sample source and tissue type.

Using TPM data from TCGA and microarray data from GSE183795, the samples were categorized into high hypoxia scoring groups and low hypoxia scoring groups based on the optimal cut-off value for hypoxia scoring obtained from survival analysis. Normalized data were analyzed for differences using the Wilcoxon rank sum test.

### 2.3 scRNA-seq data processing

Use the Seurat (version 5.0.0) package to merge all samples into the original Seurat object (26). The object is filtered according to the following parameters, removing unqualified cells: 1) doublets; 2) cells with less than 100 and more than 9,000 expressed genes; 3) cells with more than 125,000 unique molecular identifiers captured; 4) cells with more than 25% of mitochondrial genes; 5) cells with more than 50% of ribosomal genes; 6) cells with more than 5% of hemoglobin genes. Then data was log normalized. Principal component analysis was then performed. Data sets from different samples were integrated using the “Harmony” package (27). Cellular profiles were visualized by uniform manifold approximation and projection (UMAP).

Clusters were determined using the “FindClusters” function (resolution = 0.2), and identified 21 clusters of cells, which were annotated by recognized marker genes into 9 categories (epithelial cells, fibroblasts, mast cells, myeloid cells, dendritic cells, acinar cells, T&NK cells, B cells, others). After distinguishing myeloid cell subpopulations based on the marker gene (**Supplementary Table S3**) for macrophages and monocytes, macrophages were clustered at 0.1 resolution, identifying 5 clusters of cells that were annotated into tumor-associated macrophages (TAM) 1-like and TAM2-like cells by the marker gene (**Supplementary Table S3**) provided by He et al. (28) Fibroblasts were clustered at a resolution of 0.8, identifying 14 cell clusters, which were annotated into three categories of cells, the antigen-presenting fibroblasts (apCAFs), inflammatory fibroblasts (iCAFs), and myofibroblasts (myCAFs), using marker genes (**Supplementary Table S3**) provided by Elyada et al. and Affo et al. (29, 30) Epithelial cells were clustered at a resolution of 1, identifying 22 clusters, which were annotated as tumor cells and normal cells using recognized marker genes (**Supplementary Table S3**). Based on MSigDB database (https://www.gsea-msigdb.org/gsea/msigdb), 8 pancreatic cancer characteristic pathways were screened (Stem Proliferation, TGFß Signaling, Inflammatory Response, EMT, KRAS Signaling, Apoptosis, Immune Evasion) and extracted the gene set. The GSVA package was used to score the pathways. Finally, the difference in pathway activity between tumor and normal epithelial cells was compared to verify the annotation results of epithelial cells.

### 2.4 Pathway/gene set studies

The pathway/gene set scoring calculation is based on the “PROGENy” and “irGSEA” packages. The 14 pathway scores for JAK-STAT, NFκB, TNFα, Hypoxia, MAPK, EGFR, WNT, p53, TGFβ, Trail, VEGF, Androgen, Estrogen and PI3K were calculated using the package “PROGENy” (**Figure 1A**) (31). The scoring of the ferroptosis resistance gene set was calculated using the “irGSEA” package. Ferroptosis suppression genes were obtained from FerrDb V2 (http://www.zhounan.org/ferrdb/current/) after literature screening (**Supplementary Table S4**). Enrichment analysis of differential genes was performed using the WikiPathways enrichment analyses in the “clusterProfiler” package (32).

### 2.5 Construction of a WSIs-based model for predicting hypoxia levels

The tumor hypoxia score was calculated based on the RNA-seq data from TCGA-PAAD. Samples were stratified into high-hypoxia and low-hypoxia groups according to the threshold associated with the most significant prognostic value. For each group of samples, the corresponding H&E-stained pathological WSIs were extracted. Each slide was then labeled based on the hypoxia status of the sample, categorizing sections into either the high-hypoxia group or the low-hypoxia group.

Due to the huge amount of data in WSIs, there is the problem of difficult labelling and training. So, we used a method which extends attention-based multiple-instance aggregation to general multi-class weakly supervised WSIs classification, named clustering-constrained-attention multiple-instance learning (CLAM) (33). This method does not need manual region-of-interest (ROI) extraction, pixel/patch-level labelling, or naive sampling.

The CLAM workflow includes tissue segmentation and patch extraction: generating patch coordinate files through threshold segmentation: $S\left(x,y\right)=\;\{\begin{array}{c} 1\;if\;I\left(x,y\right)\;\tau_{seg}\\ 0\;otherwise \end{array}$ and morphological closing operation: $S_{closed}=\;S\;\circ \;B$ followed by feature extraction: encoding image patches using pretrained models (ResNet50/UNI) to generate 1024-dimensional feature vectors $(f_{ij}=Encoder\left(P_{ij}\right)$ during weakly supervised learning (CLAM Core), generating pseudo-labels via attention mechanisms: $a_{ij}=softmax\left(W_{a}h_{ij}+\;b_{a}\right)$ and clustering constraints based on the top 8 high-attention features, while optimizing the model with a combined cross-entropy loss and SmoothTop1 SVM loss: $ℒ=\;\lambda {ℒ}_{CE}+\left(1-\lambda \right){ℒ}_{SmoothTop1SVM}$ finally producing slide-level predictions and heatmaps.

According to the flow of the framework, we split the slide into patches of pixel size 256×256 in an equivalent pyramid of 40x magnification. Since the hue, saturation and lightness of HSV are more suitable for human color perception characteristics, we transform the patch from RGB color space to HSV color space (**Figure 1B**). According to the principle of multi-instance learning, all patches after segmentation of a patient are considered as a bag. In order to reduce the training time as well as to perform the dimensionality reduction of the data, we use the Transfer Learning and Convolutional Neural Networks approaches to transform each patch into vectors of size 1024 respectively using 2 pre-training models, ResNet50 and UNI (34, 35). The dataset was separated into training set, validation set and test set in the ratio of 8:1:1 according to the category hierarchy. According to the default settings of CLAM, model performance was tested in the 10% validation set after training the model in the training set, and 10-fold Monte Carlo cross-validation was used. The final model evaluation was performed on 10% of the test set. The loss function used smooth top-1 SVM loss (36). The parameters of the models were updated using the Adam optimizer, with the learning rate set to 2×10-4, and the weight decay set to 1×10-5. All models were trained for at least 50 epochs, or up to 200 epochs if the early stopping criterion was not met. The criterion for Early stopping was to stop this training when there was no loss reduction in the current 20 epochs. The model was saved when it has the lowest loss on the test set. In order to explain the relative importance of different regions in the slides to the final predictions of the model, we also calculated and saved the un-normalized attention scores. These attention scores were converted to percentile scores, scaled to 0 and 1.0 and visualized.

### 2.6 Immune infiltration studies

To achieve comprehensive characterization of the tumor immune microenvironment, we integrated transcriptomic signatures with pathological imaging data, constructing a multi-scale immune infiltration analysis framework across molecular, tissue, and cellular levels. This approach fully leverages the molecular quantification strengths of RNA-seq data and the spatial resolution capabilities of Whole Slide Images (WSIs).

Immune cell subtyping based on transcriptomic features was systematically analyzed using the CIBERSORT algorithm (37). Raw RNA-seq data underwent standardized processing, including CPM transformation and log2 normalization. Deconvolution analysis was then performed using the LM22 immune signature matrix, which contains gene expression profiles specific to 22 immune cell subtypes. Batch effects and technical variations were effectively eliminated through constrained least-squares regression and quantile normalization strategies. To evaluate the reliability of deconvolution results, we conducted 1,000 permutation tests to calculate confidence intervals, ultimately obtaining proportion scores of immune cell subpopulations in tumor samples.

To establish spatial correlations for molecular features, we implemented a tumor-infiltrating lymphocyte (TIL) region identification pipeline based on WSInfer (8). WSIs were first segmented into 256×256-pixel tiles at 20× magnification, with blank background regions filtered via thresholding to retain valid tissue areas. A pre-trained ResNet-50 deep learning model was then employed to extract pathological features and classify each tile, identifying TIL-enriched regions while computing confidence scores. This process generated high-resolution spatial density heatmaps of TILs. The workflow not only validated CD8^+^ T cell abundance trends observed in CIBERSORT-based molecular quantification but also provided spatial anchors for subsequent cellular-level HoVer-UNet analysis, enabling cross-scale associations from macroscopic tissue localization to microscopic cellular phenotypes.

For WSI-identified TIL hotspot regions, we performed cellular-resolution multi-modal analysis using a knowledge distillation-optimized HoVer-UNet model. By distilling knowledge from the high-performance yet computationally intensive HoVerNet model, HoVer-UNet achieved comparable or superior nuclear segmentation and classification accuracy while significantly improving inference speed (38, 39). The core architecture of HoVer-UNet integrates U-Net with a Mix Vision Transformer backbone network, enabling simultaneous capture of local detail features and global contextual information. To achieve precise multi-class nuclear identification, HoVer-UNet incorporates multiple decoder output branches for predicting nuclear semantic segmentation, horizontal/vertical distance maps, and nuclear subtypes. Selected regions of interest (ROIs) were analyzed using a HoVer-UNet model pre-trained on the PanNuke dataset, which distinguishes diverse cell types (40). This pre-training strategy effectively utilizes prior knowledge from large-scale datasets to enhance model generalization and classification accuracy for specific ROIs.

The tiered “molecular-tissue-cellular” analytical framework progresses from CIBERSORT-derived global immune signatures to WSInfer-based identification of immune-active hotspots, and finally to HoVer-UNet-enabled resolution of tumor-immune spatial heterogeneity at single-cell precision. CIBERSORT provides comprehensive immune profiling, WSInfer spatially maps immune activity patterns, and HoVer-UNet deciphers cellular interactions, collectively overcoming the limitations of single-omics methods in spatial resolution or molecular depth. This integrative strategy enables multi-scale exploration of immune microenvironment dynamics through complementary data modalities.

### 2.7 Drug sensitivity predicting

The CTRP2 dataset from the “oncoPredict” package was used as a training set to predict the IC50 of the drugs in the TCGA-PAAD dataset and the GSE183795 dataset samples (41). The drugs associated with oxygen species (ROS) (darinaparsin, BRD−K94991378, BRD−K71935468) and ferroptosis (erastin, 1S,3R−RSL−3, ML162, ML210) were selected for analysis to compare the correlation between SQOR and drug IC50.

### 2.8 H&E staining

Tissue samples were first fixed in formalin, paraffin-embedded and sectioned continuously. Then the tissue sections were obtained after dewaxing, rinsing, staining with hematoxylin and 2% eosin, dehydrating, clearing, and cover-slipping. Sections were then examined under optical microscope and scanned by Digital Pathology Slide Scanner (KF-PRO-005, KFBIO, China).

### 2.9 Immunohistochemistry staining

Tissue samples were formalin-fixed, paraffin-embedded and sectioned continuously. Target sections were dehydrated, dewaxing, antigen repaired and sealed. Sections were incubated with SQOR primary antibody (Abcam Cat# ab272574, RRID: AB_3095529) overnight at 4°C, and then followed by secondary antibody incubation at 37 °C. Diaminobenzidine was used to develop the color and counterstained with hematoxylin. Sections were then examined under optical microscope and scanned by Digital Pathology Slide Scanner (KF-PRO-005, KFBIO, China).

Immunohistochemical scores were assessed independently by 2 pathologists unrelated to the study using a double-blind method. Negative and weak positive were considered low expression, while positive and strong positive were considered high expression. We also used Qupath software for visualization of cell staining (42). It can somewhat differentiate the cellular regions in IHC-WSIs and label cells that stain positively for IHC.

### 2.10 Cell culture

Human PC cell lines (BxPC3 and PANC1) were purchased from Wuhan Pricella Biotechnology Company Limited (Pricella, China). The cells were cultured in high glucose Dulbecco’s modified Eagle medium (DMEM; 11995065, Gibco, USA) containing 10% fetal bovine serum (FBS; 10099141, Gibco, USA) and 1% double antibiotics (penicillin-streptomycin mixture; 15140122, Gibco, USA), and incubated at 37°C with 5% CO2 in a culture box. Hypoxia incubation was performed at 94% N2, 5% CO2 and 1% O2.

### 2.11 Transfection

Lentiviral packaged shRNA targeting SQOR for SQOR knockdown, as well as the control lentiviral empty vector, are both purchased from Shanghai Genechem Company Limited (GeneChem, China). Cells were plated and cultured in complete medium for 24h. The cells were infected with infection enhancer P by diluting the infection enhancer P at a ratio of complete medium: infection enhancer P of 24:1 before infection. Then the original medium of the cells was discarded, and the cells were washed with PBS. The virus was diluted to a titer of 1x108 TU/mL with complete culture medium, and the volume of virus to be added was calculated based on the MOI value. 5 μL of viral fluid was added, followed by infection for 16 h, then replaced with complete culture medium, and incubated for another 48 h. Infection efficiency was observed approximately 72 h after infection.

### 2.12 Cell proliferation assay

Cell viability was measured using the Cell Counting Kit-8 (CCK-8; Beyotime, China). Cells were inoculated into 96-well plates and 100 µl of 5000 cells were added to each well. According to the experimental needs, BxPC3 and PANC1 cells were treated under hypoxic conditions and stimulated with 5 μM erastin (HY-15763, MCE, China) and 2 mM sulfasalazine (SAS; HY-14655, MCE, China), respectively, for 24 h before sample collection and analysis. PANC1 and SQOR knockdown PANC1 cells were treated under hypoxic conditions and stimulated with 5 μM erastin and 1 μM ferrostatin- (Fer-1) (HY-100579, MCE, China), respectively, for 24 h before sample collection and analysis. 10 μL of CCK8 reagent was added to each well, incubated in a cell culture incubator at 37°C for 1 h in the dark, and then measured the absorbance at 450 nm with microplate reader (WD-2102B, LIUYI, China).

### 2.13 Transwell migration assay

The migration ability of the cells was assessed using the Transwell migration assay. The transfected cells were resuspended with medium containing 1% FBS, and the cell suspension was diluted to 3×105 cells/mL and plated into Transwell chambers (3422, Costar, USA). The lower layer was filled with complete culture medium containing 20% FBS. After 24 h of incubation under hypoxic conditions, the cells were fixed with 4% formaldehyde solution for 10 min and then stained with 0.5% crystal violet solution for 30 min. Observed under a 200× microscope to count the number of cells in each field of view.

### 2.14 Malondialdehyde content assay

Malondialdehyde (MDA) Content Assay Kit (BC0025, Solarbio, China) was used to detect MDA content according to the manufacturer’s instructions. The cells were broken down using ultrasonic waves with 1 mL of extraction solution per 5 million cells, and then centrifuged for 10 min at 4°C. Then 300 μL of MDA test solution, 100 μL of samples, and 100 μL of reagent III were added. After mixing and incubating for 60 min in a water bath at 100°C, the mixture was cooled in the ice bath and centrifuged for 10 min at room temperature. The supernatants were collected, and the absorbance of each sample was measured at 532 nm and 600nm. The MDA content was calculated based on the protein concentration.

### 2.15 Western blot analysis

Cell lysate was prepared, and cells were lysed on ice for 30 min, followed by centrifugation at 4°C for 10 min. The supernatant was taken to obtain protein samples. Protein quantification was performed using the BCA protein concentration kit (P0010, Beyotime, China), and protein concentration was calculated. The protein samples were mixed and centrifuged, then heat denatured for 10 min, and separated on SDS-PAGE gels according to the molecular weight of the target proteins. The separated proteins were transferred to a PVDF membrane. The membrane was closed with skimmed milk and incubated with primary antibody and then with secondary antibody. SQOR antibody was purchased from Proteintech Group (17256-1-AP, Proteintech, USA). GAPDH was used as a reference gene.

### 2.16 Real-time quantitative reverse transcription polymerase chain reaction assay

RNA was extracted using TRIzol (15596-018, Invitrogen, USA) and reverse transcription was performed with RT SuperMix for qPCR (K1074, APExBIO, USA). SQOR and GAPDH were then quantified using 2X SYBR Green qPCR Master Mix (K1070, APExBIO, USA). The PCR cycling conditions were as follows: pre-denaturation (hold; 1 cycle): 95°C for 2 min; 40 cycles of denaturation (95°C for 15 s), annealing (60°C for 30 s) and extension (60°C for 30 s), followed by 1 cycle of 95°C for 15 s, 60°C for 1 min and 95°C for 15 s.

SQOR:F 5′-AAGGTTTTTGCTGCGCCAAC-3′;R 5′-ATAATGGTTCCTGGCCGCAT-3′.

GAPDH:F 5′-CTCGCTTCGGCAGCACA-3′;R 5′-AACGCTTCACGAATTTGCGT-3′.

### 2.17 Animal studies

The design and implementation of this experiment was reviewed and approved by the Laboratory Animal Ethics Committee of Wenzhou Medical University (Approval number: wydw2024-0136). 4–5-week-old male Balb/c.nude mice were provided by SPF (Beijing) biotechnology Co., Ltd. The mice were 5 mice per group and were randomly and equally divided into 4 groups. Mice were subcutaneously inoculated with PANC1 cells suspended in saline. The cell density was 2x106 cells/100μL. One week later when the tumor volume reached approximately 50 mm3, the drugs were administered with DMSO control, Erastin, HTS07545 (HY-144439, MCE, China), and Erastin plus HTS07545. The long (a) and short (b) axes of the tumor were measured every 3 days (tumor volume = 1/2*a*b2). Tumor growth curves were plotted based on tumor volume. Animals were executed on reaching the humane endpoint or experimental terminative indicator, and then tumors were stripped and weighed on an analytical balance. The weighed tumors were immersed in 10% formaldehyde for the preparation of tissue sections and pathological analysis.

### 2.18 Statistical analysis

Statistical analyses were performed using R (version 4.2.2) (https://www.r-project.org/). The Wilcoxon rank sum test was used to compare differences between the two groups. The Kruskal-Wallis test was used to compare differences between the three groups. For experimental assessments, such as RT-qPCR assay and cell proliferative capacity assay, Student’s t-test was used to calculate statistical significance. The McNemar test was used for the paired four-table test. The Kaplan-Meier (KM) method was used to construct survival curves to assess prognosis. The survival distribution of the sample was tested by Log-rank test. Cox regression analysis was used to assess the effect of specific factors on patient prognosis. Spearman correlation analysis was used for correlation analysis. P < 0.05 was considered statistically significant.

## 3 Results

### 3.1 Hypoxia as an oncogenic driver in malignant tumors

**Figure 2:**
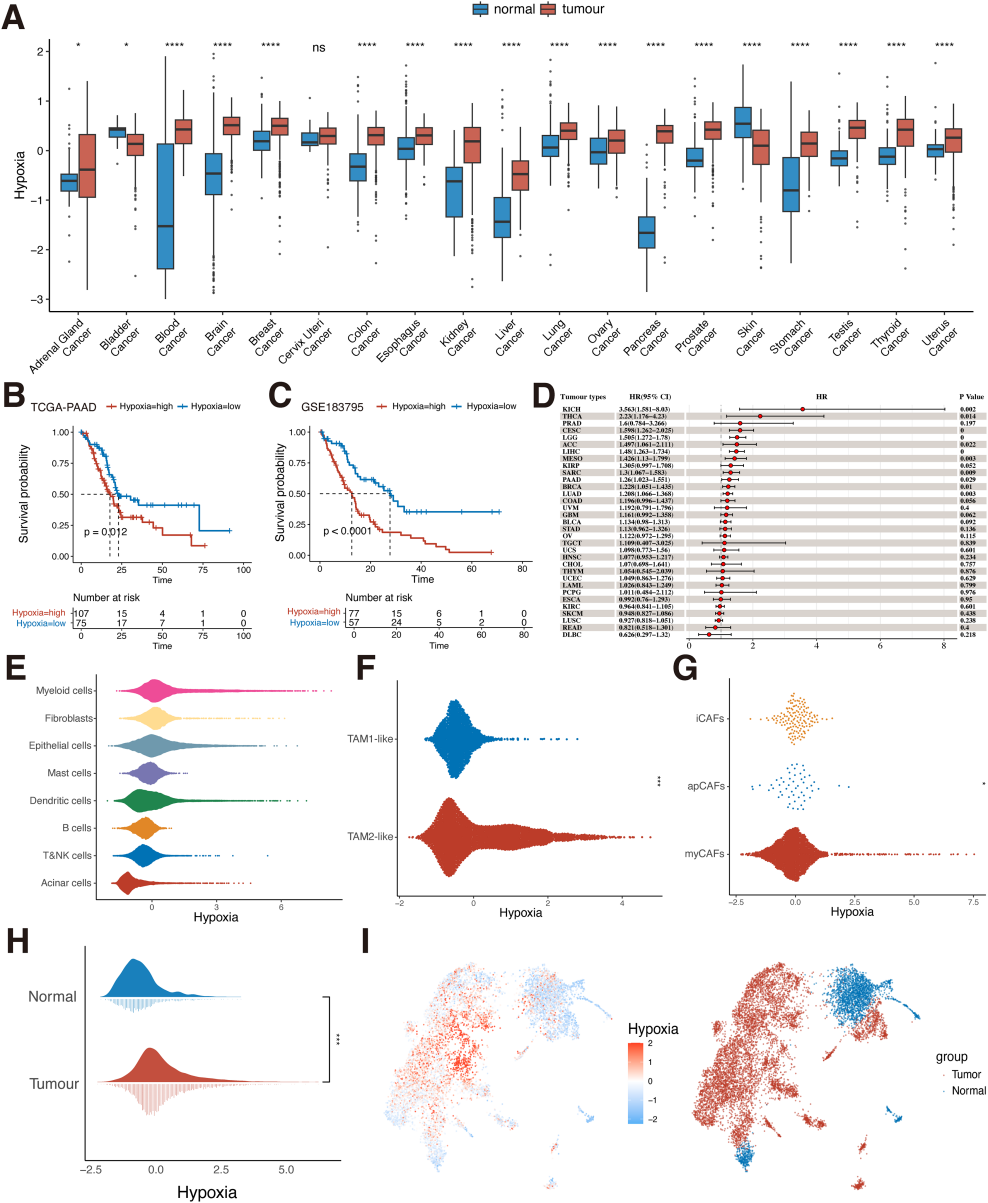
Hypoxia level in TME and prognosis. **(A)** Differences in the distribution of hypoxia in normal and tumor tissues in the bulk dataset at the pan-cancer level (Wilcoxon rank sum test). **(B)** KM survival analysis of hypoxia for the TCGA-PAAD dataset (Log-rank test). **(C)** KM survival analysis of hypoxia for the GSE183795 dataset (Log-rank test). **(D)** Univariate Cox analysis of hypoxia in pan-cancer. **(E)** Hypoxia scoring of eight cell types at the single-cell level (Wilcoxon rank sum test). **(F)** Hypoxia scoring of TAM1-like and TAM2-like subpopulations (Wilcoxon rank sum test). **(G)** Hypoxia scoring of apCAFs, iCAFs and myCAFs subpopulations (Wilcoxon rank sum test). **(H)** Differences in the distribution of hypoxia in normal and tumor cells in pancreatic epithelial cells at single-cell level (Wilcoxon rank sum test). **(I)** UMAP plot of hypoxia scoring in pancreatic normal and tumor epithelial cells. ns, P≥ 0.05; *, P<0.05; ***, P<0.001; ****, P<0.0001. TCGA, The Cancer Genome Atlas; KM, Kaplan-Meier; UMAP, uniform manifold approximation and projection; KICH, Kidney Chromophobe; THCA, Thyroid carcinoma; PRAD, Prostate adenocarcinoma; CESC, Cervical squamous cell carcinoma and endocervical adenocarcinoma; LGG, Brain Lower Grade Glioma; ACC, Adrenocortical carcinoma; LIHC, Liver hepatocellular carcinoma; MESO, Mesothelioma; KIRP, Kidney renal papillary cell carcinoma; SARC, Sarcoma; PAAD, Pancreatic adenocarcinoma; BRCA, Breast invasive carcinoma; LUAD, Lung adenocarcinoma; COAD, Colon adenocarcinoma; UVM, Uveal Melanoma; GBM, Glioblastoma multiforme; BLCA, Bladder Urothelial Carcinoma; STAD, Stomach adenocarcinoma; OV, Ovarian serous cystadenocarcinoma; TGCT, Testicular Germ Cell Tumors; UCS, Uterine Carcinosarcoma; HNSC, Head and Neck squamous cell carcinoma; CHOL, Cholangiocarcinoma; THYM, Thymoma; UCEC, Uterine Corpus Endometrial Carcinoma; LAML, Acute Myeloid Leukemia; PCPG, Pheochromocytoma and Paraganglioma; ESCA, Esophageal carcinoma; KIRC, Kidney renal clear cell carcinoma; SKCM, Skin Cutaneous Melanoma; LUSC, Lung squamous cell carcinoma; READ, Rectum adenocarcinoma; DLBC, Lymphoid Neoplasm Diffuse Large B-cell Lymphoma; TAM, tumor-associated macrophages; apCAFs, antigen-presenting fibroblasts; iCAFs, inflammatory fibroblasts; myCAFs, myofibroblasts.

Hypoxia has now been shown to be present in most solid tumors and is considered a hallmark of cancer (43). The highly malignant nature of pancreatic cancer is largely attributed to the hypoxic TME (10). We first downloaded the GTEX dataset and selected the corresponding 28 TCGA primary tumor datasets by organ origin for hypoxia scoring calculation. The results showed that in most tumor tissues (e.g. pancreatic, liver, kidney, colon, and stomach cancers) hypoxia scoring was significantly higher than that in normal tissues (p<0.05, **Figure 2A**). However, in bladder cancer and melanoma, hypoxia scoring was higher in normal tissue than in tumor tissue. The results of the pan-cancer analysis tentatively confirmed the high hypoxia levels in various types of tumors. Moreover, it is worth noting that in PDAC, the hypoxia score of tumor tissues was much higher than that of normal tissues (0.271 ± 0.438 vs. -1.85 ± 1.63, P<0.0001, **Figure 2A**). Meanwhile, PDAC patients with high hypoxia levels had a worse prognosis (p < 0.05, **Figures 2B, C**). However, this is not limited to PDAC alone. We further used univariate Cox analysis to evaluate the prognostic impact of hypoxia in tumor patients. We found that hypoxia was a prognostic risk factor in a variety of tumors including pancreatic cancer (kidney chromophobe, lung adenocarcinoma, lower grade glioma, etc.) (p < 0.05, **Figure 2D**). This suggests that hypoxia is a common characteristic of malignant tumors and a potential factor driving carcinogenesis.

We used single-cell data from pancreatic cancer for analysis, identifying eight known cell types including epithelial cells, myeloid cells, fibroblasts, acinar cells, dendritic cells, mast cells, B cells, T and NK cells (**Supplementary Figure S1**). To assess the level of hypoxia in the TME of PDAC, we scored hypoxia in all cell types (**Figure 2E**). The results showed higher levels of hypoxia scoring in three cell types: myeloid cells, fibroblasts, and epithelial cells. This implies that these cell types may play an important role in helping PDAC construct a suitable survival hypoxic TME. Therefore, three cell types, myeloid cells, fibroblasts, and epithelial cells, were selected for further analysis. We identified and extracted macrophages from myeloid cells and broadly categorized them into two macrophage subpopulations, TAM1-like and TAM2-like, based on the classical TAM-related marker gene (**Supplementary Figure S2A**). TAMs are the main component of immune cells in the TME, and different subtypes of TAMs have different functions (44). M1 macrophages exert anti-tumor capabilities. M2 macrophages are the main manifestation phenotype of TAMs and promote the occurrence and development of tumors. We found that the TAM2-like subpopulation had significantly higher hypoxia scoring than the TAM1-like subpopulation (P<0.05, **Figure 2F**). This is similar to previous findings that hypoxia promotes macrophage polarization in the TME in a direction that favors tumor progression, exhibiting high hypoxia levels (45). We then further analyzed the scoring of hypoxia in fibroblasts. The apCAFs, iCAFs, and myCAFs subpopulations were first identified (**Supplementary Figure S2B**) and scored for hypoxia. The results showed that there was a difference in hypoxia scoring among the three subpopulations (P<0.05, **Figure 2G**), with the iCAFs subpopulation exhibiting the highest hypoxia scoring among the three subpopulations. This suggests that hypoxia-related pathways are overactivated in iCAFs. Previous studies have also identified that iCAFs are mainly enriched in the hypoxic regions of PDAC tumors and participate in the malignant progression of PDAC (46). Finally, we extracted the epithelial cells and classified them simply by classical marker gene into tumor cells and normal cells (**Supplementary Figure S2C**), and verified them by tumor-specific pathway score (**Supplementary Figure S2D**). It can be seen that in tumor cells, the hypoxia score showed a difference compared with normal cells (P<0.05, **Figures 2H, I**). It indicates that hypoxia in various cells may be involved in the construction of the immunosuppressive tumor ecosystem and promote the malignant progression of PDAC cells.

### 3.2 MIL-based hypoxia discrimination model construction and model interpretation

**Figure 3:**
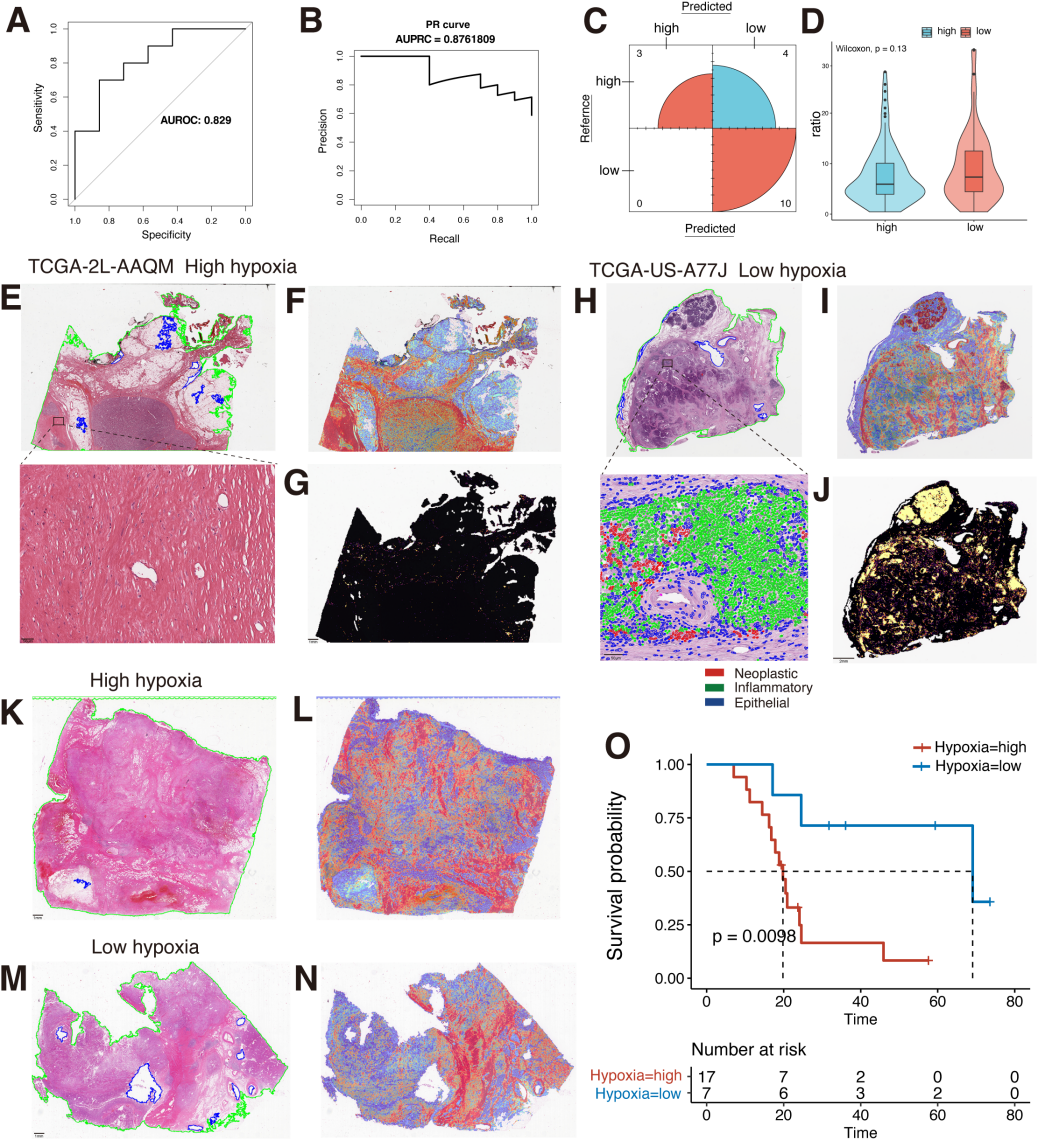
MIL-based WSIs hypoxia differentiation model construction. **(A)** The AUROC for the hypoxia detection model is 0.829. **(B)** The AUPRC is 0.876. **(C)** Confusion matrix in the test set. **(D)** Comparison of the ratio of tiles testing positive for TILs to full tiles in the two groups of high and low hypoxia (Wilcoxon rank sum test). **(E)** Original pathological images of TCGA-2L-AAQM sample and magnified views of high-weight regions. **(F)** Model interpretation heatmap for the TCGA-2L-AAQM sample. **(G)** Spatial distribution of TILs in the TCGA-2L-AAQM sample (yellow: TIL-positive regions). **(H)** Original pathological images of TCGA-US-A77J sample and magnified views of high-weight regions. **(I)** Model interpretation heatmap for the TCGA-US-A77J sample. **(J)** Spatial distribution of TILs in the TCGA-US-A77J sample (yellow: TIL-positive regions). **(K)** Original pathological image of a clinical sample classified as hypoxia-high by the model. **(L)** Model identification as low hypoxia by heatmap interpretation of clinical samples. **(M)** Original pathological image of a clinical sample classified as hypoxia-low by the model. **(N)** Model identification as low hypoxia by heatmap interpretation of clinical samples. **(O)** Analysis of KM survival in high and low hypoxia groups in a clinical sample. MIL, multi-instance learning; WSIs, Whole Slide Images; TCGA, The Cancer Genome Atlas; TILs, tumor-infiltrating lymphocytes; AUROC, the area under the curve of the receiver operating characteristic; AUPRC, the area under the curve of the precision-recall; ROI, region-of-interest; KM, Kaplan-Meier.

The importance of WSIs has been largely ignored in previous studies of PDAC patient characteristics, in part because the huge resolution of WSIs presents unique computational and methodological challenges (33). In recent years, with the development of AI technology, advances in deep learning for computational pathology have enabled WSIs to be used for automated cancer diagnosis and quantification of morphological phenotypes in the TME. Therefore, in order to determine whether WSIs can provide assistance in differentiating patients’ hypoxia levels and further help clinicians to judge the prognosis of PDAC patients according to WSIs, we performed patients’ hypoxia level detection based on the CLAM framework proposed by Lu et al. (33) The results showed that the hypoxia detection model we constructed could effectively identify the hypoxia level of patients, with an AUROC of 0.829, an AUPRC of 0.876, and an accuracy of 0.7647 (**Figures 3A–C**). We also compared recently published UNI models that showed excellent performance in pre-training of pathology images but did not show better performance in our task due to our small sample size and the large risk of overfitting (**Supplementary Figure S3**). We further applied this model to our clinical PDAC samples, and the results showed that patients who were considered by the model to have high hypoxia levels had a significantly poorer prognosis (**Figure 3O**), which indicated that the model could effectively identify the hypoxia levels of tissues and had a better prospect for clinical application and promotion.

To better interpret the constructed hypoxia discrimination model, we used heatmap to visualize the attentional weight scores in the last layer of the model. Both in the TCGA dataset and in our clinical samples, the high weight regions of the model in samples predicted to have high hypoxia levels were broadly focused on the tumor stroma. In contrast, in samples predicted to have low hypoxia levels, the model’s areas of attention were predominantly lymphoid tissue and tumor stroma (**Figures 3E–N**). This suggests that hypoxia levels in tumor tissue can be observed in clinical WSIs, which provides some basis for subsequent patient prognosis identification based on interpretable pathology. Due to the different regions in the identification of high and low hypoxia levels in the models, and the TME analysis based on TCGA transcriptome data, it was also suggested that the hypoxia level in tumor tissue was negatively correlated with the level of CD8^+^ T cell infiltration in the TME in various tumors, including PDAC (**Supplementary Figure S4**). Therefore, we further explored the correlation between hypoxia levels and lymphocytes in WSIs. We used the “WSInfer” framework to identify the TILs region in WSIs (8). The results also showed that the proportion of TILs was lower in samples with high hypoxia scores than in samples with low hypoxia (**Figures 3E–J**). However, when comparing the overall ratios of the two groups, no statistically significant difference was shown, despite the high hypoxia group having a higher overall ratio than the low hypoxia group (**Figure 3D**). In part, this may be due to the fact that quantity-based TILs comparisons do not reflect cell status and cell type in TILs (47, 48). We also used the HoVer-UNet framework to identify cell types at high weight regions in patients with low hypoxia, which further suggests that the model did observe some differences in the level of immune infiltration in tissues with high and low levels of hypoxia. This indicates that the hypoxia-related features in WSIs are effectively identified by the model. However, in general, hypoxia plays an important role in the construction of the tumor immunosuppressive microenvironment, which can be directly reflected in WSIs, and the combined with a deep learning method has the potential to characterize or quantify hypoxia levels at the clinical level.

### 3.3 SQOR may be involved in the progression of PDAC in a hypoxic microenvironment

**Figure 4:**
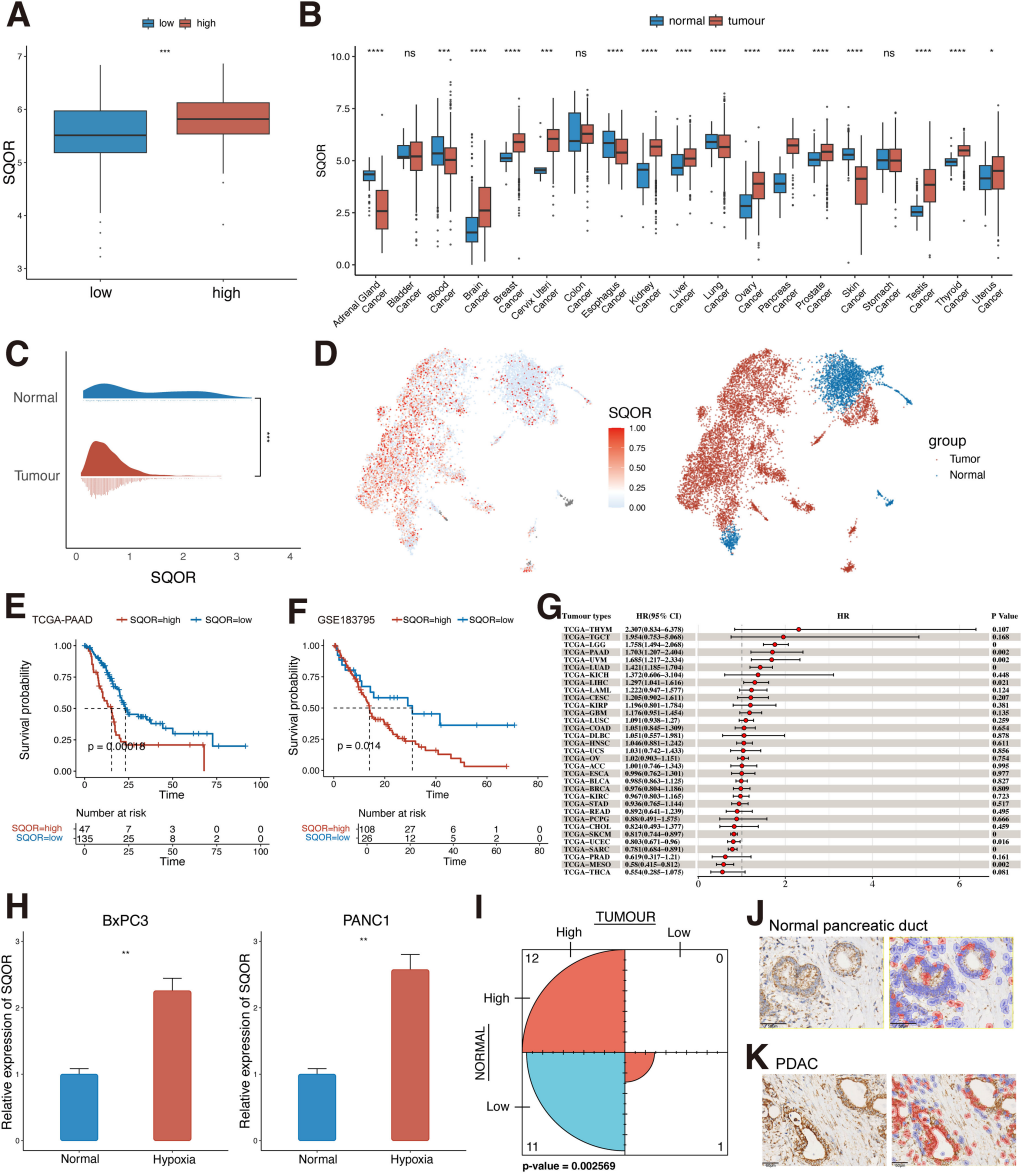
SQOR may be involved in PDAC progression in a hypoxic microenvironment. **(A)** Differences in the distribution of SQOR between low and high hypoxic tissues in the TCGA-PAAD dataset (Wilcoxon rank sum test). **(B)** Differences in the distribution of SQOR in normal and tumor tissues in the bulk dataset at the pan-cancer level (Wilcoxon rank sum test). **(C)** Differences in the distribution of SQOR in pancreatic normal epithelial and tumor cells at the single-cell level (Wilcoxon rank sum test). **(D)** UMAP plot of SQOR in normal and tumor epithelial cells of the pancreas. **(E)** KM survival analysis of SQOR for the TCGA-PAAD dataset (Log-rank test). **(F)** KM survival analysis of SQOR for the GSE183795 dataset (Log-rank test). **(G)** Univariate Cox analysis of SQOR in pan-cancer. **(H)** RT-qPCR results of SQOR of BxPC3 and PANC1 cells under normal and hypoxia conditions (Student’s t-test). **(I)** Counting results of SQOR IHC staining of tumor samples and normal samples from clinical samples (McNemar test). **(J)** SQOR IHC staining results of normal pancreatic ductal tissue. **(K)** SQOR IHC staining results of PDAC (red: SQOR IHC-positive cells, blue: SQOR IHC-negative cells). ns, P≥ 0.05; *, P<0.05; **, P<0.01; ***, P<0.001; ****, P<0.0001. PDAC, Pancreatic ductal adenocarcinomas; TCGA, The Cancer Genome Atlas; KM, Kaplan-Meier; IHC, immunohistochemistry; RT-qPCR, Real-time quantitative reverse transcription polymerase chain reaction assay; KICH, Kidney Chromophobe; THCA, Thyroid carcinoma; PRAD, Prostate adenocarcinoma; CESC, Cervical squamous cell carcinoma and endocervical adenocarcinoma; LGG, Brain Lower Grade Glioma; ACC, Adrenocortical carcinoma; LIHC, Liver hepatocellular carcinoma; MESO, Mesothelioma; KIRP, Kidney renal papillary cell carcinoma; SARC, Sarcoma; PAAD, Pancreatic adenocarcinoma; BRCA, Breast invasive carcinoma; LUAD, Lung adenocarcinoma; COAD, Colon adenocarcinoma; UVM, Uveal Melanoma; GBM, Glioblastoma multiforme; BLCA, Bladder Urothelial Carcinoma; STAD, Stomach adenocarcinoma; OV, Ovarian serous cystadenocarcinoma; TGCT, Testicular Germ Cell Tumors; UCS, Uterine Carcinosarcoma; HNSC, Head and Neck squamous cell carcinoma; CHOL, Cholangiocarcinoma; THYM, Thymoma; UCEC, Uterine Corpus Endometrial Carcinoma; LAML, Acute Myeloid Leukemia; PCPG, Pheochromocytoma and Paraganglioma; ESCA, Esophageal carcinoma; KIRC, Kidney renal clear cell carcinoma; SKCM, Skin Cutaneous Melanoma; LUSC, Lung squamous cell carcinoma; READ, Rectum adenocarcinoma; DLBC, Lymphoid Neoplasm Diffuse Large B-cell Lymphoma.

In view of our findings suggesting that hypoxia is associated with the malignant progression of PDAC, we further analyzed the potential factors that may be involved in hypoxia-promoted carcinogenesis in PDAC (**Figure 1C**). The hypoxia score threshold with the best prognostic ability divided TCGA-PAAD patients into high hypoxia score group and low hypoxia score group for differential analysis. The results showed that a large number of genes changed between the two groups. Among them, SQOR showed significant differences between groups (**Figure 4A**). To further characterize the significance of SQOR, we used RT-qPCR *in vitro* experiments to detect the level of SQOR expression in PDAC cells under hypoxic conditions, and as we guessed, SQOR expression was up-regulated in BxPC3 and PANC1 cells cultured under hypoxic environment compared with normal culture conditions (P<0.01, **Figure 4H**). This implies that SQOR may promote the survival of pancreatic cancer cells in the harsh hypoxic microenvironment.

Analysis of SQOR expression levels in normal and tumor tissues of the pancreas revealed that SQOR was upregulated in tumor tissues and was significantly different from normal tissues (p<0.05, **Figure 4B**). The same results were obtained at the single cell level in pancreatic normal and tumor cells (**Figures 4C, D**). Meanwhile, KM survival curves suggested that patients with high SQOR expression had a worse prognosis (p < 0.05, **Figures 4E, F**). And further subgroup analyses of patients suggested that the prognosis of high hypoxia scoring and high SQOR expression subgroup was significantly lower than that of low hypoxia scoring and low SQOR expression subgroup (p<0.01, **Supplementary Figures S5A, B**, **Supplementary Table S5**). Multivariate cox analysis showed that SQOR was an independent risk factor for prognosis in PDAC (p<0.05, **Supplementary Table S6**). The effect of SQOR in pan-cancer on the prognosis of tumor patients was further explored, and the results showed that SQOR was a prognostic risk factor in PDAC, liver hepatocellular carcinoma, brain lower grade glioma, lung adenocarcinoma, and uveal melanoma (UVM) (**Figure 4G**). Proteomic analysis revealed that SQOR was up-regulated in tumor epithelial-enriched cores and bulk tissues, but did not differ in tumor stroma-enriched cores (**Supplementary Figure S5C**). IHC results in clinical patients similarly showed significantly higher staining intensity of SQOR in tumor tissues compared to normal tissues (P<0.05, **Figures 4I–K**). Combined with bulk and single-cell transcriptomics, proteomics, and clinical IHC data, SQOR is up-regulated in tumors, and its high expression strongly predicts poor prognosis of patients. This suggests that SQOR under hypoxic conditions plays a crucial role in driving the malignant progression of PDAC.

### 3.4 Spatial transcriptomics and pathomics suggest co-localization of hypoxia and SQOR expression

**Figure 5:**
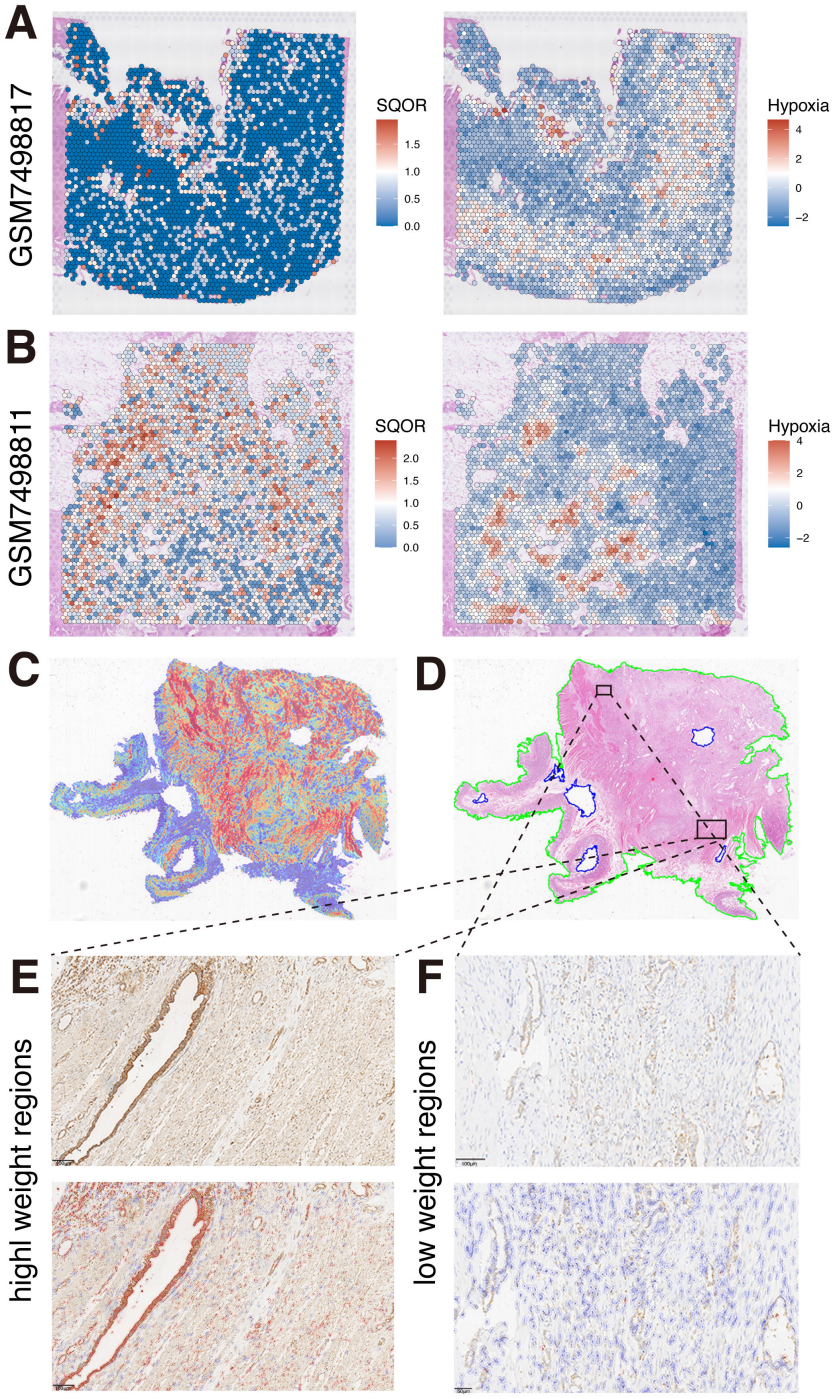
Spatial co-localization of hypoxia and SQOR expression. **(A)** Spatial localization of SQOR and hypoxia in GSM749817 samples. **(B)** Spatial localization of SQOR and hypoxia in GSM749811 samples. **(C)** Heatmap of clinical samples. **(D)** Original pathological images of clinical samples. **(E)** SQOR IHC staining results of high attention weighted regions (red: SQOR IHC-positive cells, blue: SQOR IHC-negative cells). **(F)** SQOR IHC staining results of low attention weighted regions (red: SQOR IHC-positive cells, blue: SQOR IHC-negative cells). IHC, immunohistochemistry.

Based on our multi-omics study, a strong correlation between hypoxia and SQOR was suggested and confirmed by our *in vitro* experiment. We further investigated whether hypoxia and SQOR expression are linked in tissue space. We used the dataset from GSE235315 for further studies. By visualizing the spatial distribution of hypoxia score and SQOR at the single-cell level, we found that regions with high hypoxia score were accompanied by high expression of SQOR (**Figures 5A, B**, **Supplementary Figure S6**). This suggests that there is a spatial level co-localization of the hypoxia pathway with SQOR expression. It also suggests that there is heterogeneity in hypoxia at the spatial level and that this heterogeneity is accompanied by altered SQOR expression. We also observed our clinical WSIs. Given that the high-attention regions in our hypoxia-expressing explanatory clinicopathological heatmap were mainly focused on the stromal region of the tumor, but the weight of stromal attention varied among different regions. This suggests that the hypoxia model also paid attention to the structural differences in the stroma (**Figure 5C**). Previous studies have shown that the high hypoxia in PDAC tissues is partly due to the lack of vascularity of the tumor tissue and the high stromal levels (49). Meanwhile, clinical SQOR IHC results of WSIs corresponding to HE pathology showed that SQOR was strongly positive in stromal wrapped ductal malignant epithelial cells with high attention weight (**Figure 5D**). Interestingly, we observed weaker SQOR staining in ductal malignant cells encapsulated by stroma at relatively low weight regions than in the former (**Figures 5E, F**). This further suggests a spatial co-localization of hypoxia and SQOR expression.

### 3.5 Hypoxia induces ferroptosis resistance in PDAC

**Figure 6:**
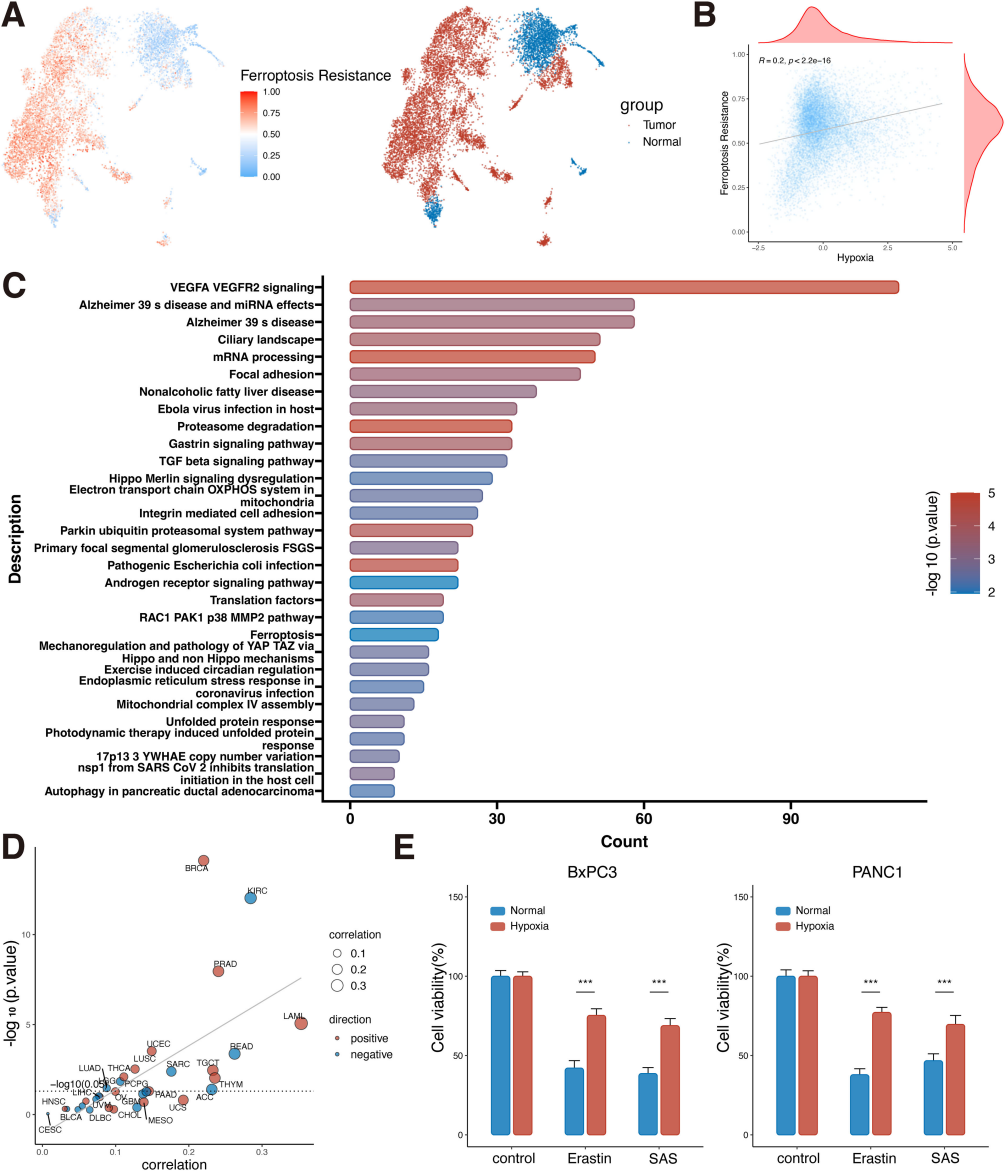
Ferroptosis resistance in PDAC cells under hypoxic conditions. **(A)** UMAP plot of ferroptosis resistance scoring in pancreatic normal and tumor epithelial cells. **(B)** Correlation analysis of hypoxia and ferroptosis resistance at the single-cell level (Spearman correlation). **(C)** Differential gene wikipathway enrichment analysis of tumor and normal epithelial cells at the single-cell level. **(D)** Correlation analysis of hypoxia and ferroptosis resistance in the bulk dataset at the pan-cancer level (Spearman correlation). **(E)** Cell viability assay of BxPC3 and PANC1 cells under hypoxic and normal conditions plus erastin or SAS (Student’s t-test). ***, P<0.001. PDAC, Pancreatic ductal adenocarcinomas; UMAP, uniform manifold approximation and projection; KICH, Kidney Chromophobe; THCA, Thyroid carcinoma; PRAD, Prostate adenocarcinoma; CESC, Cervical squamous cell carcinoma and endocervical adenocarcinoma; LGG, Brain Lower Grade Glioma; ACC, Adrenocortical carcinoma; LIHC, Liver hepatocellular carcinoma; MESO, Mesothelioma; KIRP, Kidney renal papillary cell carcinoma; SARC, Sarcoma; PAAD, Pancreatic adenocarcinoma; BRCA, Breast invasive carcinoma; LUAD, Lung adenocarcinoma; COAD, Colon adenocarcinoma; UVM, Uveal Melanoma; GBM, Glioblastoma multiforme; BLCA, Bladder Urothelial Carcinoma; STAD, Stomach adenocarcinoma; OV, Ovarian serous cystadenocarcinoma; TGCT, Testicular Germ Cell Tumors; UCS, Uterine Carcinosarcoma; HNSC, Head and Neck squamous cell carcinoma; CHOL, Cholangiocarcinoma; THYM, Thymoma; UCEC, Uterine Corpus Endometrial Carcinoma; LAML, Acute Myeloid Leukemia; PCPG, Pheochromocytoma and Paraganglioma; ESCA, Esophageal carcinoma; KIRC, Kidney renal clear cell carcinoma; SKCM, Skin Cutaneous Melanoma; LUSC, Lung squamous cell carcinoma; READ, Rectum adenocarcinoma; DLBC, Lymphoid Neoplasm Diffuse Large B-cell Lymphoma.

Various previous studies have shown that solid tumors can be resistant to ferroptosis in the hypoxic microenvironment, but the regulatory mechanism is still unclear (23). Based on the above results of high hypoxia level in PDAC, we further used single-cell data to evaluate the ferroptosis resistance level in PDAC epithelial cells, and the data analysis showed that the ferroptosis resistance score of tumor cells was much higher than that of normal cells (P<0.05, **Figure 6A**). At the same time, WP pathway enrichment analysis of tumor cells and normal epithelial cells showed that tumor cells had changes in VEGF signaling pathway, focal adhesion, proteasome degradation, TGFβ signaling pathway, and ferroptosis pathway (**Figure 6C**). Meanwhile, the single-cell level analysis of PDAC showed a positive correlation between hypoxia and ferroptosis resistance (R=0.2, P<0.001, **Figure 6B**). As previously reported, the link between hypoxia and ferroptosis resistance is also likely to exist in a variety of tumors (23).

Pan-cancer analysis at the bulk level found a positive correlation between hypoxia and ferroptosis resistance in a variety of tumors, such as PDAC, breast cancer, prostate cancer, and lung squamous cell carcinoma (P<0.05, **Figure 6D**). Therefore, it is speculated that PDAC can promote tumor survival by inhibiting ferroptosis under harsh hypoxic environment. To confirm our hypothesis, *in vitro* experiments were performed to simulate the tumor microenvironment of PDAC cells with high hypoxia and high ferroptosis pathway activation. CCK8 assay was used to detect the anti-ferroptosis ability of PDAC cells under hypoxic environment with Erastin and SAS inducers. BxPC3 and PANC1 cells cultured under hypoxia were more resistant to ferroptosis inducers than those cultured under normal culture conditions (P<0.05, **Figure 6E**). This result confirms the strong resistance of tumor cells to ferroptosis under hypoxic conditions.

### 3.6 High SQOR expression promotes ferroptosis resistance of PDAC cells in hypoxic microenvironment

**Figure 7:**
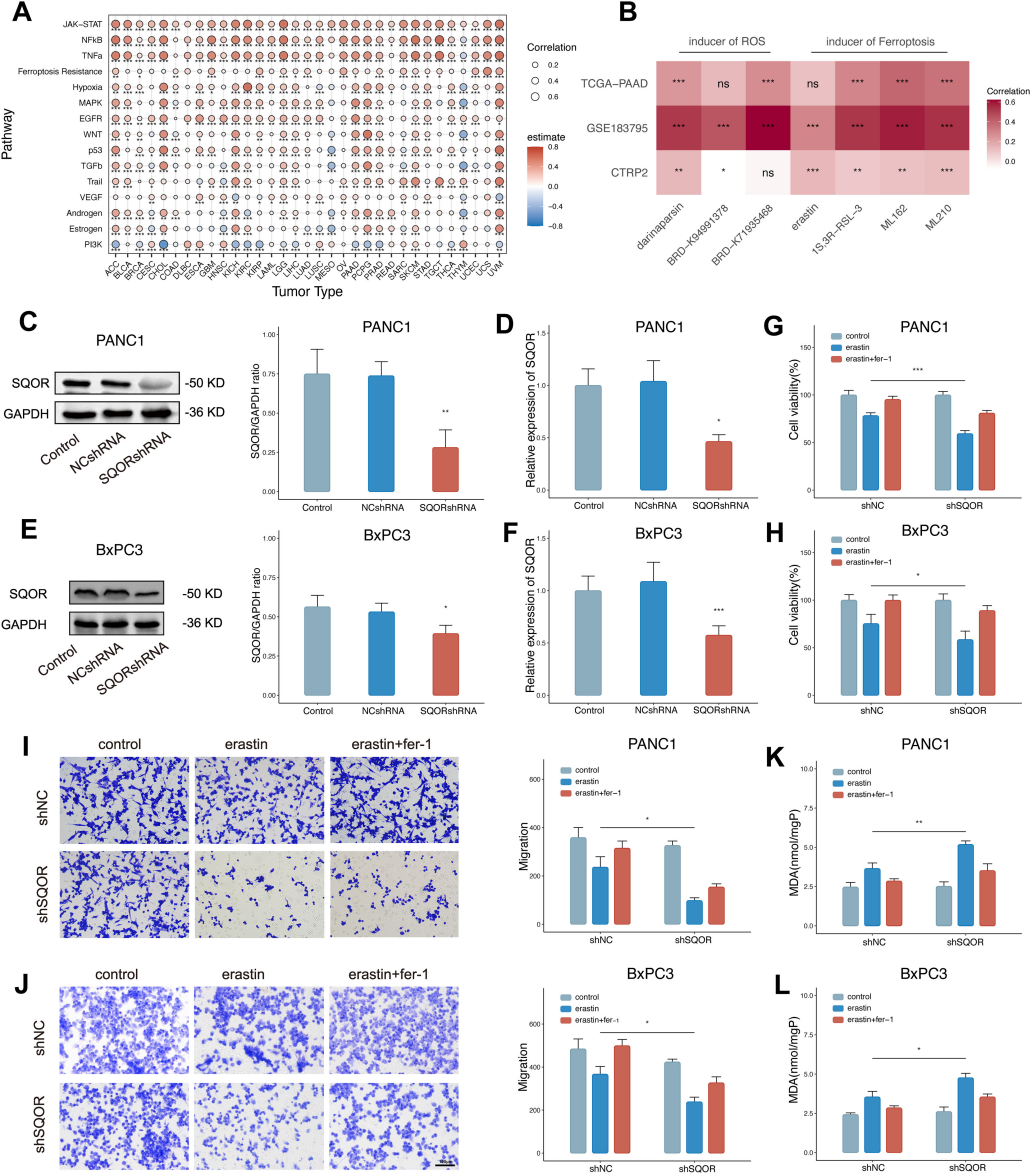
High SQOR expression promotes ferroptosis resistance of PDAC cells in hypoxic microenvironments. **(A)** Correlation analysis of SQOR with multiple pathways/genes set scores including hypoxia and ferroptosis resistance in the bulk dataset at the pan-cancer level (Spearman correlation). **(B)** IC50 correlation analysis of SQOR with ROS inducers (darinaparsin, BRD-K94991378, BRD-K71935468) and ferroptosis inducers (erastin, 1S,3R-RSL-3, ML162, ML210) (Spearman correlation). **(C)** The SQOR knockdown PANC1 cell line was validated by western blot (Student’s t-test). **(D)** SQOR knockdown PANC1 cell line validated by RT-qPCR (Student’s t-test). **(E)** The SQOR knockdown BxPC3 cell line was validated by western blot (Student’s t-test). **(F)** SQOR knockdown BxPC3 cell line validated by RT-qPCR (Student’s t-test). **(G)** Cell viability assay of SQOR knockdown and non-knockdown PANC1 cells in control, plus erastin and plus erastin+Fer-1 groups (Student’s t-test). **(H)** Cell viability assay of SQOR knockdown and non-knockdown BxPC3 cells in control, plus erastin and plus erastin+Fer-1 groups (Student’s t-test). **(I)** Cell migration capacity assay of SQOR knockdown and non-knockdown PANC1 cells in control, plus erastin and plus erastin+Fer-1 groups (Student’s t-test). **(J)** Cell migration capacity assay of SQOR knockdown and non-knockdown BxPC3 cells in control, plus erastin and plus erastin+Fer-1 groups (Student’s t-test). **(K)** MDA levels were assayed in SQOR knockdown and non-knockdown PANC1 cells in control, plus erastin and plus erastin+Fer-1 groups (Student’s t-test). **(L)** MDA levels were assayed in SQOR knockdown and non-knockdown BxPC3 cells in control, plus erastin and plus erastin+Fer-1 groups (Student’s t-test). ns, P≥ 0.05; *, P<0.05; **, P<0.01; ***, P<0.001. PDAC, Pancreatic ductal adenocarcinomas; ROS, oxygen species; RT-qPCR, Real-time quantitative reverse transcription polymerase chain reaction assay; MDA, Malondialdehyde.

**Figure 8:**
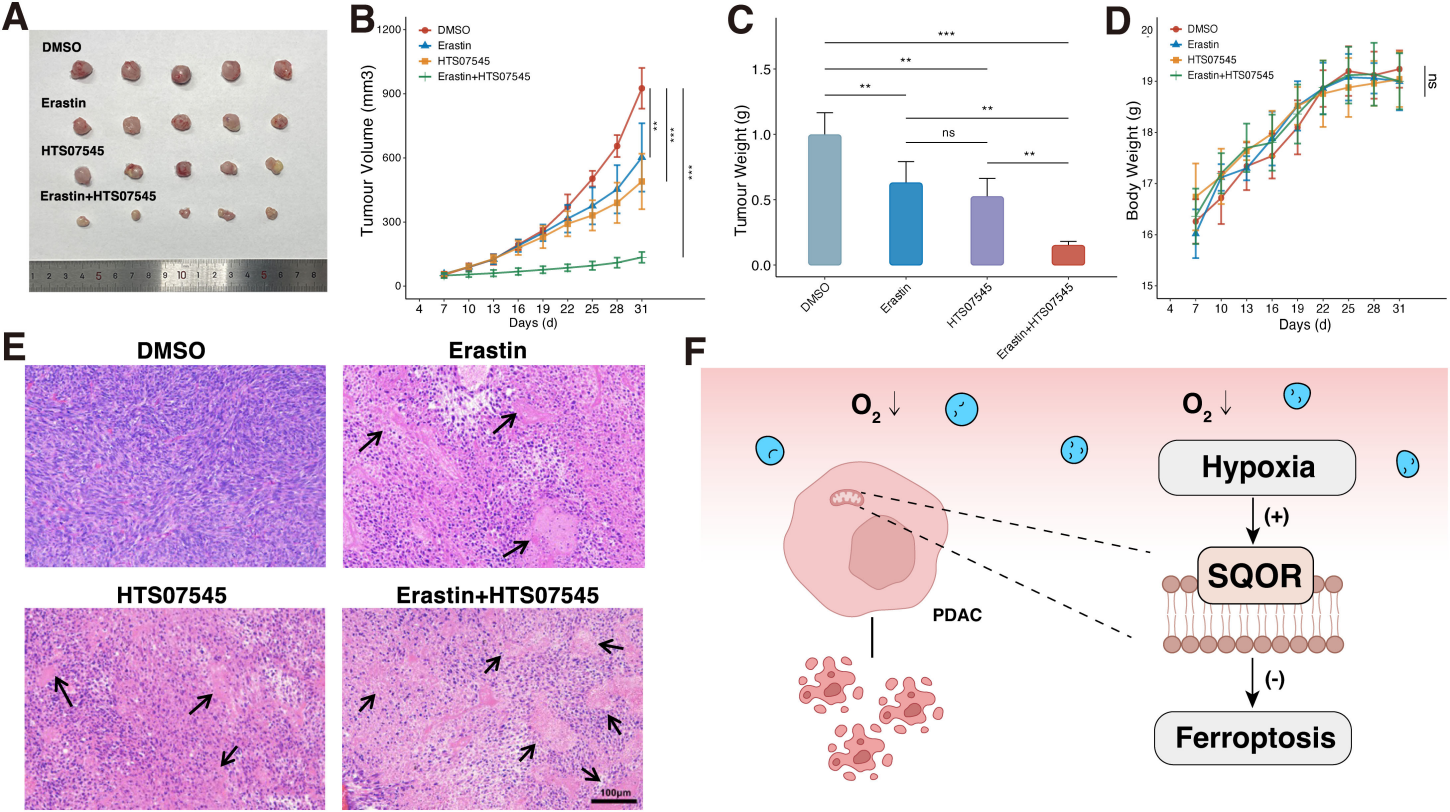
SQOR inhibitor and ferroptosis inducer have synergistic ferroptosis induction *in vivo*. **(A)** Tumor images in mice. **(B)** Tumor growth curves in mice. **(C)** Analysis of tumor weights in mice. **(D)** Body weight change curves in mice. **(E)** H&E staining of pathological sections of mice tumors. **(F)** Mechanistic diagram of the promotion of ferroptosis resistance by SQOR in PDAC cells under hypoxia. ns, P≥ 0.05; **, P<0.01; ***, P<0.001. PDAC, Pancreatic ductal adenocarcinomas.

In the bulk level pan-cancer analysis, the correlation analysis results between SQOR and 15 pathways/gene sets showed that 10 tumors (such as PDAC, kidney renal papillary cell carcinoma, prostate adenocarcinoma, uterine corpus endometrial carcinoma, UVM, etc.) were positively correlated with both hypoxia and ferroptosis resistance (**Figure 7A**). In the PDAC single-cell data, the correlation analysis results between SQOR and 15 pathway/gene set scores also showed that SQOR expression in malignant pancreatic ductal cells was positively correlated with hypoxia and ferroptosis resistance (**Supplementary Figure S7A**). Proteomic analyses also revealed a high positive correlation between SQOR and the negative regulator of ferroptosis, STAT3 (R=0.64, p=0.013) and LCN2 (R=0.73, p=0.0029), in epithelial tissue (**Supplementary Figure S7B**). Moreover, the IC50 values of tumor drugs in several datasets were calculated using the CTRP database, which showed that tumors with high SQOR expression were more resistant to ferroptosis inducers (erastin, 1S,3R-RSL-3, ML162, ML210) and ROS inducers (darinaparsin, BRD-K94991378, BRD K71935468) (**Figure 7B**). Combined with the previous bioinformatics analyses of transcriptomics and proteomics and the experimental results, it was suggested that SQOR could help PDAC tolerate the hypoxic microenvironment while making the tumor cells more resistant to ferroptosis (**Figure 8F**).

To verify this conjecture, we established stable SQOR knockdown PDAC cell lines (PANC1 and BxPC3) and verified by WB and RT-qPCR (**Figures 7C–F**). We used erastin to induce ferroptosis in pancreatic cancer cells under hypoxic conditions. After hypoxia treatment, SQOR knockdown cells showed reduced cell viability, decreased cell migration ability, and increased MDA content compared with control cells, and all the above could be reversed in the presence of Fer-1 (**Figures 7G–L**). The experimental results showed that under hypoxic conditions, SQOR could promote the resistance of PDAC cells to ferroptosis, and reduce the decreased cell viability, invasion, and oxidative damage caused by ferroptosis.

Next, we further investigated whether the SQOR inhibitor HTS07545 and the ferroptosis inducer erastin have synergistic ferroptosis-inducing effects *in vivo*. In terms of bulk specimens, the tumor volume of PDAC transplant in DMSO group was larger than that in erastin group, HTS07545 group, and erastin plus HTS07545 combined treatment group in mice (**Figure 8A**). The most significant decrease in tumor volume was observed in the combination treatment group (**Figure 8B**). Observation of tumor weights revealed that both HTS07545 and erastin groups showed a decrease in tumor weights in mice after treatment, with the most pronounced decrease in tumor weights in the erastin plus HTS07545 combination group, which had an inhibitory effect on tumor growth (**Figure 8C**). The body weight of the mice in each group was also monitored during the treatment period, and there was no significant weight loss in each group, suggesting fewer side effects (**Figure 8D**). H&E staining showed that the morphology of tumor cells in the DMSO group was normal, while the HTS07545 group and erastin group showed varying degrees of nuclear necrosis and tumor histocytological changes. Among them, the necrotic area of the erastin plus HTS07545 combined treatment group was the largest (**Figure 8E**). The results demonstrated that SQOR inhibitor HTS07545 and ferroptosis inducer erastin synergistically inhibit tumor growth *in vivo* with minimal side effects, which holds promise for clinical translation.

## 4 Discussion

PDAC is a highly malignant and lethal tumor (50). For the majority of patients with advanced PDAC, existing therapies, such as immunotherapy, chemotherapy, and radiotherapy, provide only limited clinical benefits (51). Therefore, new therapeutic options are urgently needed to improve the prognosis of patients with PDAC. Hypoxia is common in most solid tumors and its presence has been shown to increase the likelihood of cancer progression and spread (6). However, unlike other tumors, PDAC contains large numbers of stromal cells and abundant extracellular matrix (ECM), but lacks blood vessels, resulting in persistent and severe hypoxia within the tumor (49). The biology of hypoxic cancer cells is shaped by the interplay between pervasive oxygen tension, hypoxia-induced signaling pathways, interacting genetic mutations, and cellular damage caused by reactive oxygen species (ROS) (6). Furthermore, our bioinformatics analyses have identified a high hypoxia signature in a variety of solid tumors, and it is particularly evident that high hypoxia levels in PDAC are accompanied by a poor prognosis in patients. Thus, it is important to further investigate the characteristics and potential mechanisms of hypoxia in PDAC.

Our analysis of hypoxia levels in eight cell types at the single-cell level showed that myeloid cells, fibroblasts, and epithelial cells had the highest hypoxia scores. Similar to previous studies, the hypoxia score of the M1-like TAMs subpopulation was significantly higher than that of the M2-like TAMs subpopulation in macrophages. TAMs exhibit distinct functions depending on their subtypes. Specifically, M1-like TAMs primarily exert anti-tumor activity, whereas M2-like TAMs represent the predominant macrophage phenotype in PDAC, characterized by immunosuppressive and pro-tumorigenic properties (44, 52). Furthermore, studies have shown that TAM enrichment levels are higher in hypoxic regions of solid tumors and that hypoxia may be a key driver of macrophage recruitment and polarization in the TME, inducing macrophage phenotype that favor to tumor growth (53). CAFs are broadly defined as fibroblasts located in or near the tumor mass. The large number of CAFs in PDAC tissues constructs a favorable environment for tumor development (54). There is a large heterogeneity of CAFs, and the CAFs that are associated with the promotion of tumor progression are iCAFs. It was found that hypoxia in the TME of PDAC enhances the iCAFs phenotype and promotes tumor growth (46, 55). Our hypoxia scoring of CAFs also showed similar results: the iCAFs subpopulation had a higher hypoxia score than the myCAFs subpopulation. In the epithelial cell subpopulation, the hypoxia score of tumor cells was also much higher than normal. Under hypoxic conditions, PDAC achieves metabolic reprogramming through the induction of transcription factors and other methods. This process is accompanied by increased proliferation and invasive capacity of cancer cells (56). Results based on single-cell analysis showed a high hypoxic state in a variety of cells associated with tumor progression. Collectively, these findings position hypoxia as a master regulator of cellular crosstalk in the PDAC microenvironment, driving both stromal remodeling and epithelial malignancy (57).

In recent years, with the advancement of AI research and the enhancement of computer hardware capabilities, it has become possible to utilize WSIs, which contain abundant tissue information and pathological characteristics, for research purposes. The inherent tissue heterogeneity captured in WSIs provides a rich resource for tumor biology investigation, however, the manual annotation of morphologically critical regions remains labor-intensive and subjective. To address this, as a high-throughput deep learning framework, a CLAM-based method has demonstrated capabilities comparable to those of pathologists in tasks such as tumor diagnosis, tumor subtype differentiation, and patient prognosis determination, thus demonstrating their utility as scalable tools for WSIs interpretation (33, 58). However, there are fewer studies on certain characteristics of tumors at the slide level. Therefore, we used this framework to distinguish between high and low levels of hypoxia at the slide level, achieving good performance. This suggests that the level of tumor hypoxia can be directly reflected in the clinical WSIs, despite the difficult-to-explain nature of the deep learning model as a black-box model. However, to delineate biologically interpretable patterns from the model for subsequent studies, we performed model attention weight visualization. The model primarily focuses mostly on the stromal region of the tumor, as well as on the region of lymphocyte infiltration in the WSIs, but it does not pay enough attention to the epithelial region of the tumor. Previous studies have shown that the stroma of pancreatic cancer is the key stroma for disease progression (59). In addition, the histology of PDAC is characterized by massive connective tissue hyperplasia, with the resulting fibrotic reaction caused by excess fibroblasts and tumor-induced ECM deposition (60, 61). The dense ECM induces angiogenesis, hypoxia, and impairs anti-tumor immunity (62). This suggests that the model we constructed observes this feature in PDAC and evaluates hypoxia levels at the slide level based on these features in WSIs. This is further reflected in the prognostic observations of our clinical cohort. Intriguingly, while our slide-level analysis did not reveal statistically significant differences in total TILs abundance between hypoxia subgroups, we observed an inverse correlation between hypoxia levels and TIL density across multiple specimen sections. This apparent discrepancy may arise from the inability of WSI-based TIL quantification to differentiate lymphocyte subtypes or functional states. For instance, exhausted T cells tend to be enriched in tumors with impaired anti-tumor immunity (47). However, the model’s attention heat map similarly suggested a connection between hypoxia levels and lymphatic infiltration. This association was also demonstrated in the putative RNA-seq-based levels of immune cell infiltration.

Based on the widespread presence of hypoxia in PDAC cells and bioinformatics analysis, SQOR was further identified as one of the key factors in the malignant progression of tumor cells in a hypoxic TME. Comparative analysis of TCGA-PAAD cohorts revealed differential SQOR expression between high- and low-hypoxia score subgroups. Bulk RNA sequencing and single-cell transcriptomics analyses consistently demonstrated significant SQOR upregulation in tumor and normal pancreatic tissues/cells. This transcriptional pattern was corroborated at the protein level through IHC staining and proteomic profiling, confirming SQOR overexpression in PDAC specimens. Meanwhile, we also found that SQOR was an independent risk factor for the prognosis of PDAC patients. This reveals the potential of SQOR as a biomarker for PDAC. To fully utilize the WSIs information from H&E and IHC staining of our clinical cohort, we noted that the intensity of SQOR staining was significantly higher in tumor cells surrounding the region of high interest in the model compared to tumor cells in the region of relatively low interest. This feature was also evident in the spatial transcriptome data of pancreatic cancer. This suggests a spatial co-localization as well as a strong correlation between SQOR and hypoxia. To verify the guess, further *in vitro* experiments confirmed the upregulation of SQOR expression in PDAC cells cultured under hypoxic environment compared to normal conditions. SQOR is located in mitochondria and can reduce ubiquinone via the electron transport chain (63, 64). Meanwhile, ROS are generated by various enzymatic and non-enzymatic processes in the cell and are important mediators of cellular signaling (64). The production of ROS in mitochondria has been shown to be involved in hypoxia signaling. Mitochondria are a major source of cellular ROS. Increased mitochondrial proton conductance leads to the conversion of ubiquinone to ubiquinol, reducing ROS production due to oxidative stress and other factors (65). Kleiner et al. demonstrated that silencing of SQOR in wild-type HeLa cells leads to an increase in ROS (66). Thus, PDAC cells survival and proliferation under hypoxic conditions may result from increased SQOR expression through accelerated ubiquinone/ubiquinol cycling in the mitochondria. Lee et al. have indicated that intracellular SQOR under physiological conditions reduces ROS in the cell by regulating ubiquinone/ubiquinol cycling (67). Although further experimental confirmation of this mechanism in PDAC is still needed, we found that total ubiquinone/ubiquinol content in the PANC1 cell line was increased compared to normal pancreatic epithelial cell levels using metabolomics data from Yang et al., suggesting that there is an elevated ubiquinone/ubiquinol cycle level in PDAC (**Supplementary Figure S8**) (68).

As our above studies have shown that high levels of hypoxia and SQOR are present in tumor cells and differential analysis indicates alterations in the ferroptosis pathway in tumor cells. This suggests that SQOR under hypoxic conditions may affect cancer progression by modulating the ferroptosis pathway. Ferroptosis, an iron-dependent regulated cell death mechanism driven by lipid peroxidation, demonstrating significant potential in cancer therapy (18). In addition, cellular iron is critical for maintaining multiple metabolic pathways. Iron accumulation is one of the key signals initiating membrane oxidative damage during ferroptosis (69). Excess iron promotes subsequent lipid peroxidation through two mechanisms: the production of ROS and the activation of iron-containing enzymes. Tumor cell growth is significantly dependent on the trace element iron compared to non-malignant cells. Inhibition of ferroptosis promotes tumor invasion and metastasis. Bioinformatics analysis indicated a positive correlation between SQOR and both hypoxia and ferroptosis resistance in PDAC. Meanwhile, by simulating the hypoxic environment of PDAC cells and the ecological microenvironment with a high activation level of ferroptosis pathway, our *in vitro* experiments demonstrated the existence of ferroptosis resistance under hypoxic microenvironment in PDAC and this phenotype reversible by the ferroptosis inhibitor fer-1. However, the underlying mechanisms of hypoxia and ferroptosis resistance in the TME need to be further explored. It has been found that hypoxia can prevent ferroptosis by increasing the transcription of SLC7A11 and HO-1 and reducing ROS (70, 71). Our study extends the theory that hypoxia protects PDAC cells from ferroptosis by upregulating SQOR expression. This study provides a new insight into the mechanism of ferroptosis resistance of PDAC under hypoxia.

## 5 Conclusions

In conclusion, in this study, we first determined that hypoxia plays an important role in the progression of malignant tumors. As PDAC exhibits a higher level of hypoxia and a worse prognosis than most tumors, we further analyzed the potential factors involved in hypoxia-promoting carcinogenesis in PDAC. We found that SQOR was highly expressed in PDAC and was associated with poor prognosis. We then explored the relationship between hypoxia and ferroptosis and found a positive correlation between hypoxia and ferroptosis resistance in several tumors, including PDAC. *In vitro* experiments demonstrated that SQOR promotes ferroptosis resistance in PDAC cells under hypoxic conditions. *In vivo* experiments demonstrated that both the SQOR inhibitor HTS07545 and the ferroptosis inducer erastin could inhibit tumors and both have synergistic inhibitory effects on tumor growth with fewer side effects. Therefore, we suggest that the hypoxic microenvironment in PDAC is a major factor contributing to ferroptosis resistance, and SQOR is an important gene for this process. There are also limitations to our study. Firstly, there was a deficiency in the number of cells studied at our single-cell level, and the hypoxia, SQOR and ferroptosis resistance properties of PDAC were not observed in a wider dataset. Second, the hypoxic properties of PDAC were not observed in more types of TME cells. Third, due to limited public resources for matching pathological sections of pancreatic cancer samples to transcriptome data, the dataset size is small, especially for validation and test samples. Fourth, we did not perform external validation of the model in our clinical cohort, although our clinical cohort has demonstrated the prognostic significance of the constructed model. Overall, we analyzed the important role of hypoxia in PDAC from multiple dimensions (bioinformatics, computer vision, *in vitro* experiments and *in vivo* experiments). We discovered that hypoxia can be identified as a phenomenon in clinicopathological slides, while simultaneously revealing a novel mechanism of hypoxia-mediated ferroptosis resistance. Furthermore, this study underscores the potential of SQOR as both a biomarker and a therapeutic target in PDAC, thus warranting further in-depth investigation.

## Data Availability

The original contributions presented in the study are included in the article/**Supplementary Material**. Further inquiries can be directed to the corresponding author.
