# Supplementary material for: Comprehensive analysis of SQOR involvement in ferroptosis resistance of pancreatic ductal adenocarcinoma in hypoxic environments
Source: Front Immunol. 2025 May 1;16:1513589. doi: 10.3389/fimmu.2025.1513589 (PMC12078260; doi:10.3389/fimmu.2025.1513589)
Supplement: Supplementary file 1 [file DataSheet1.zip › data/Public dataset sources.docx]

| **Public dataset sources** | | | |
| --- | --- | --- | --- |
| dataset | | sample | references |
| TCGA | | n=11057 | https://gdc.cancer.gov/about-data/publications/pancanatlas |
| GTEx | | n=5172 | https://gdc.cancer.gov/about-data/publications/pancanatlas |
| GEO | GSE183795 | n=134 | PMID: 36426859 |
|  | GSE155698 | n=20 | PMID: 36411320, 34296197 |
|  | GSE235315 | n=7 | PMID: 38297291 |
