# Supplementary figures and images for: Comprehensive analysis of SQOR involvement in ferroptosis resistance of pancreatic ductal adenocarcinoma in hypoxic environments
Source: Front Immunol. 2025 May 1;16:1513589. doi: 10.3389/fimmu.2025.1513589 (PMC12078260; doi:10.3389/fimmu.2025.1513589)

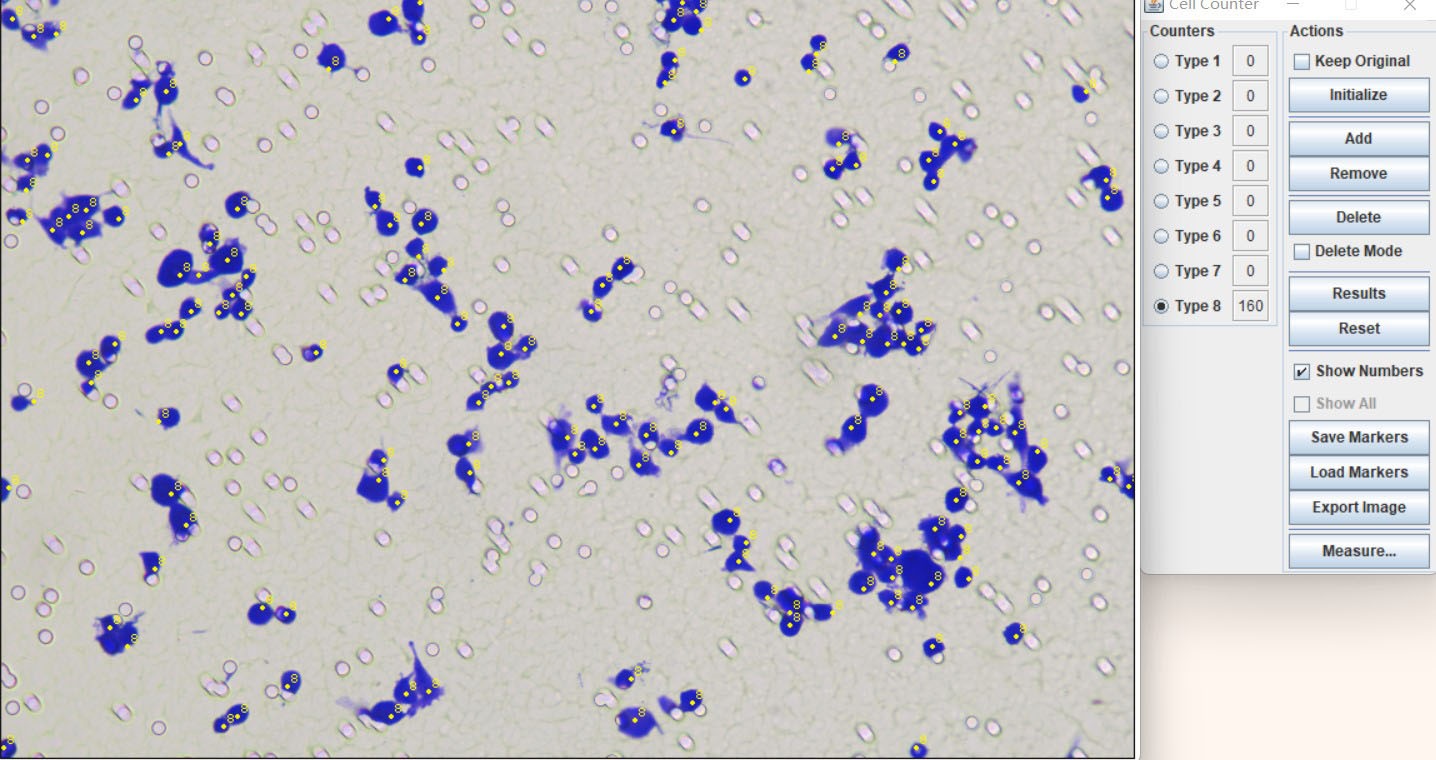

Supplement: Supplementary file 1 [file DataSheet1.zip › data/experiment/shSQOR e+f.jpg]

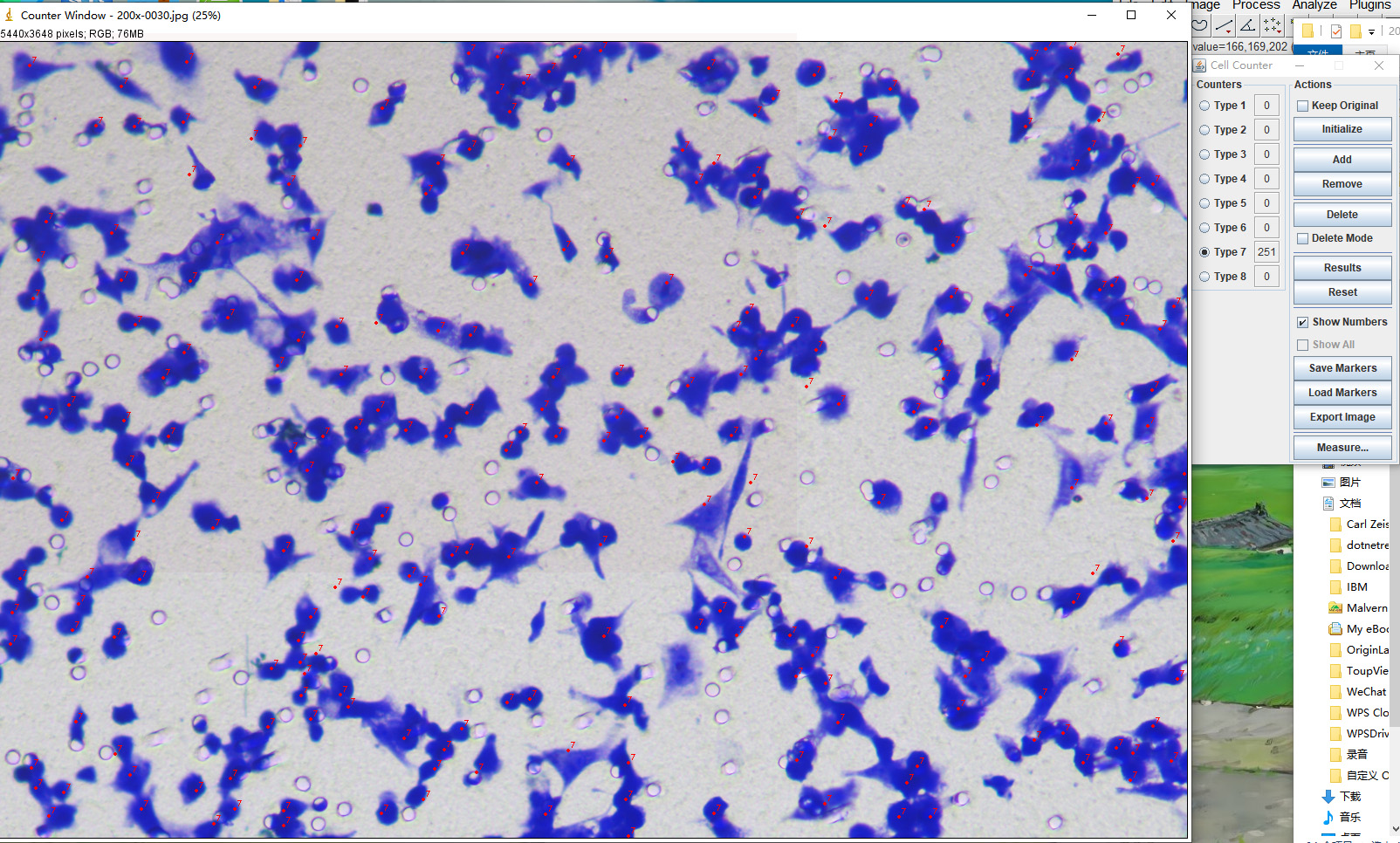

Supplement: Supplementary file 1 [file DataSheet1.zip › data/experiment/shNC erastin.jpg]

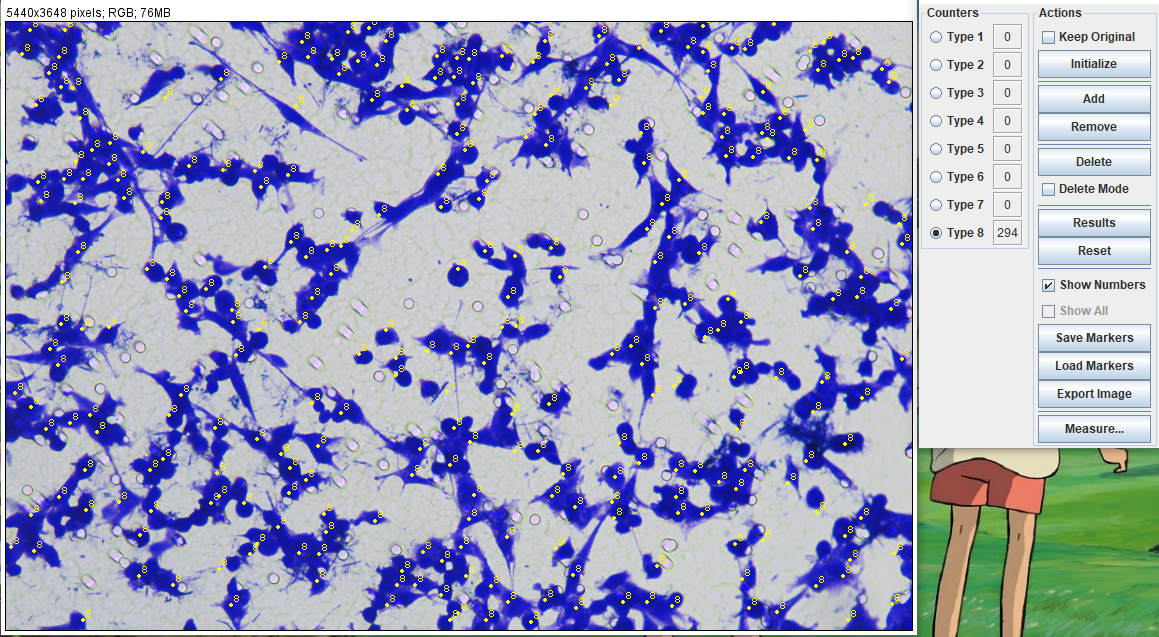

Supplement: Supplementary file 1 [file DataSheet1.zip › data/experiment/shNC e+f.jpg]

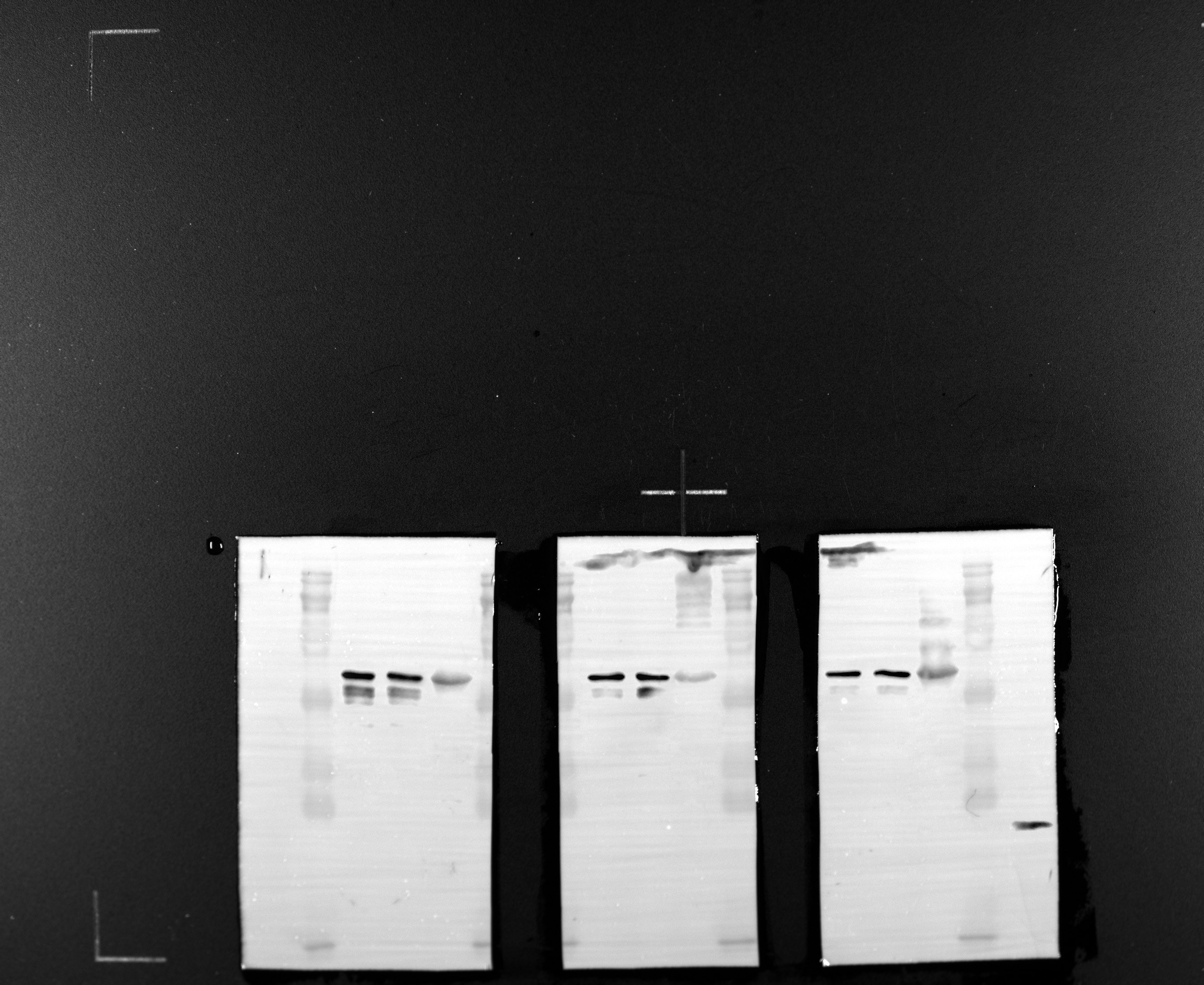

Supplement: Supplementary file 1 [file DataSheet1.zip › data/experiment/SQOR.jpg]

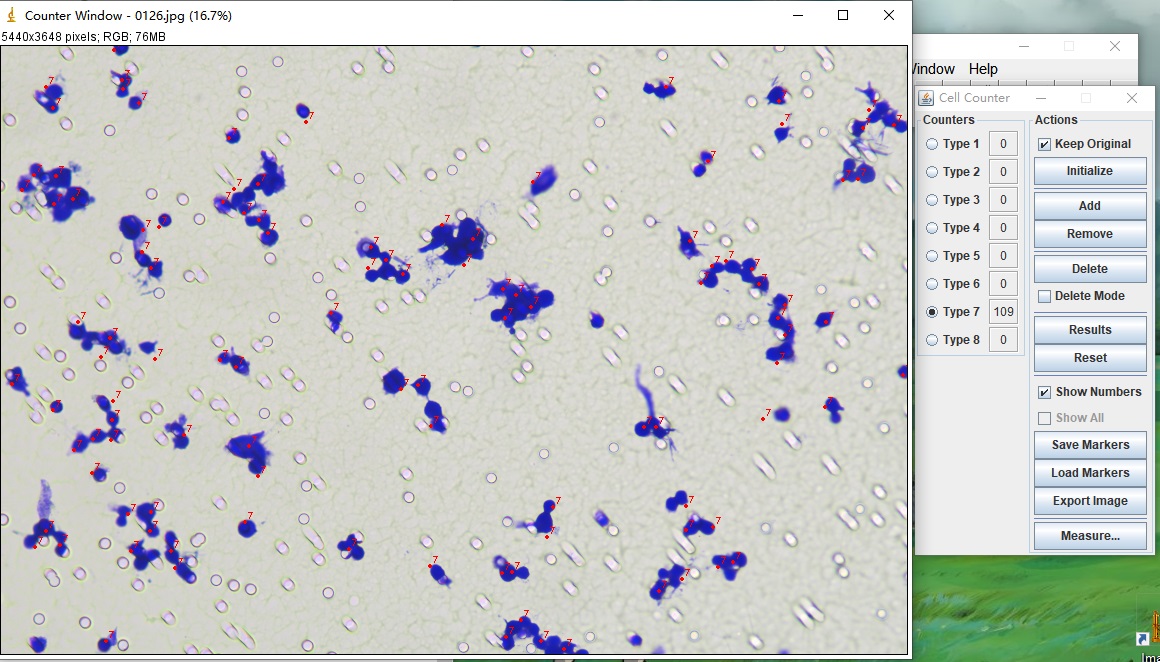

Supplement: Supplementary file 1 [file DataSheet1.zip › data/experiment/shSQOR erastin.jpg]

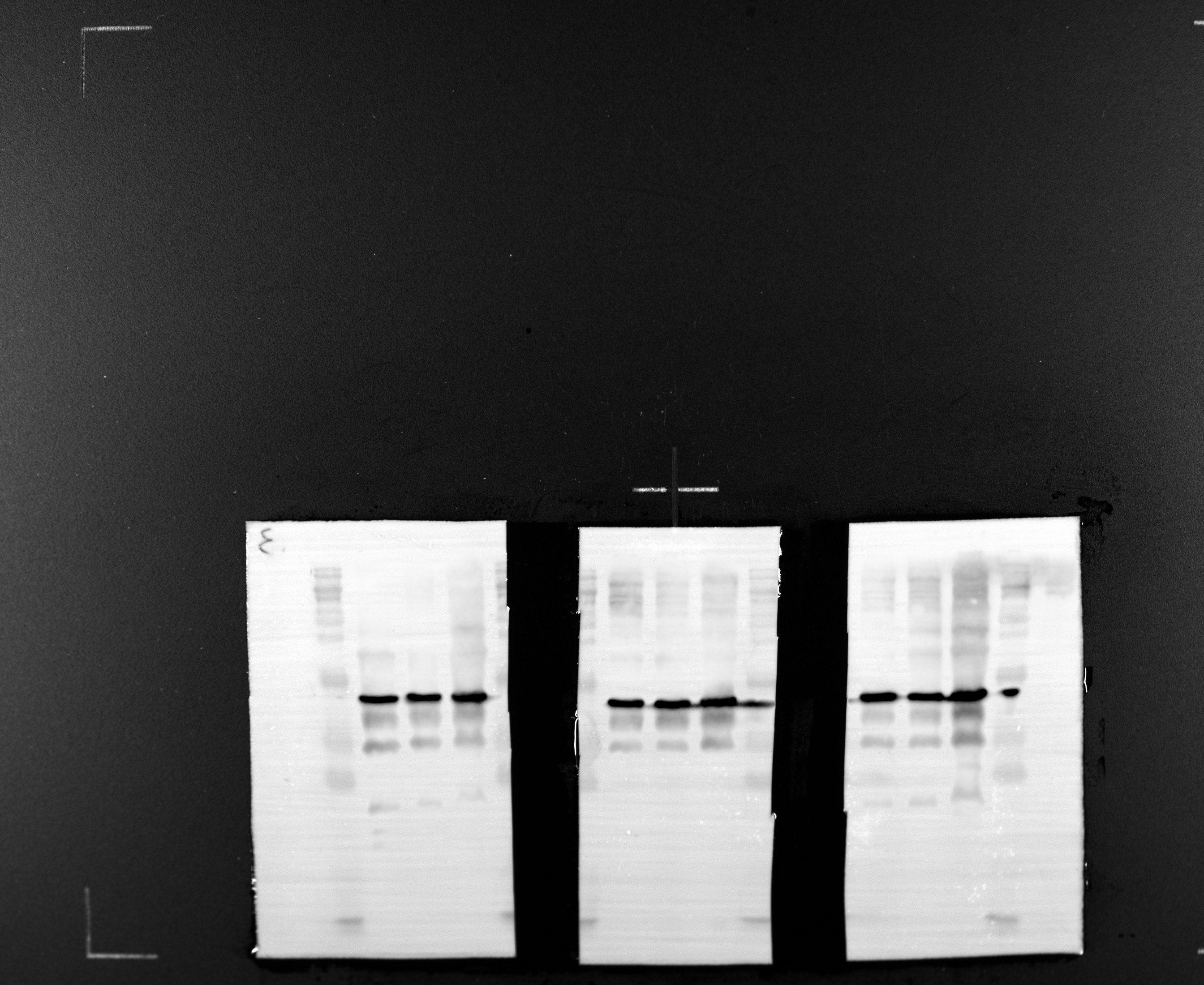

Supplement: Supplementary file 1 [file DataSheet1.zip › data/experiment/GAPDH.jpg]

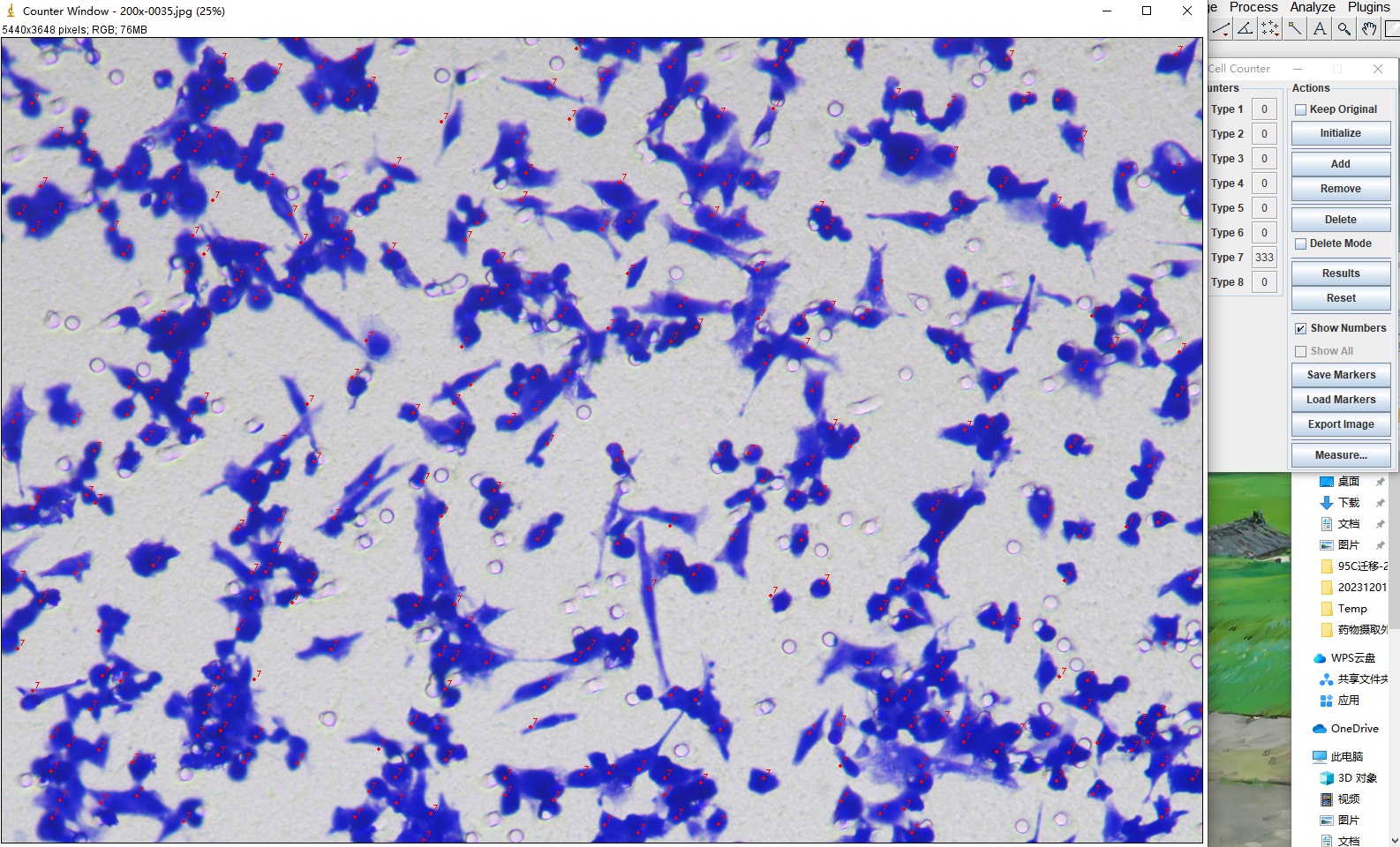

Supplement: Supplementary file 1 [file DataSheet1.zip › data/experiment/shSQOR control.jpg]

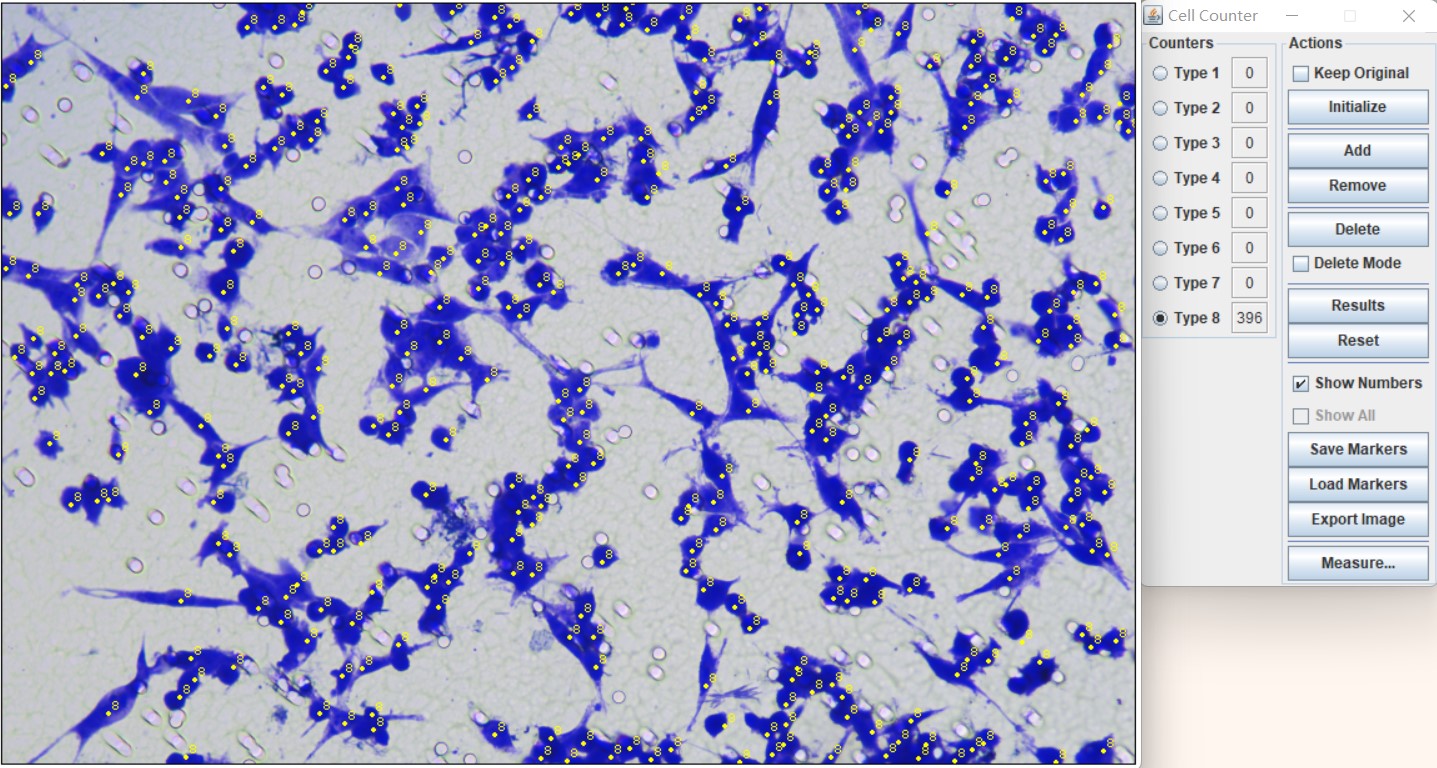

Supplement: Supplementary file 1 [file DataSheet1.zip › data/experiment/shNC control.jpg]

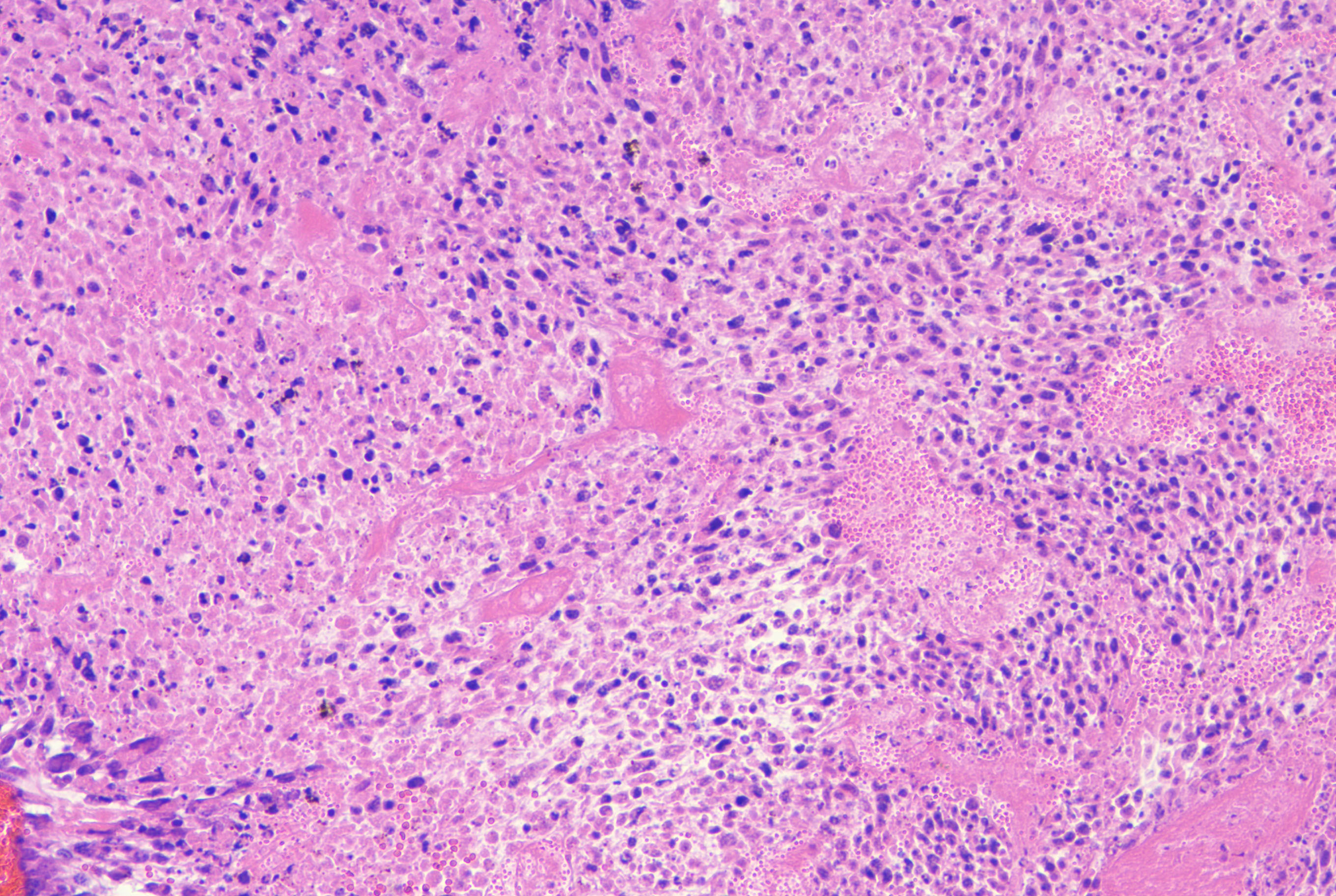

Supplement: Supplementary file 1 [file DataSheet1.zip › data/experiment/animal/E+H 200X.jpg]

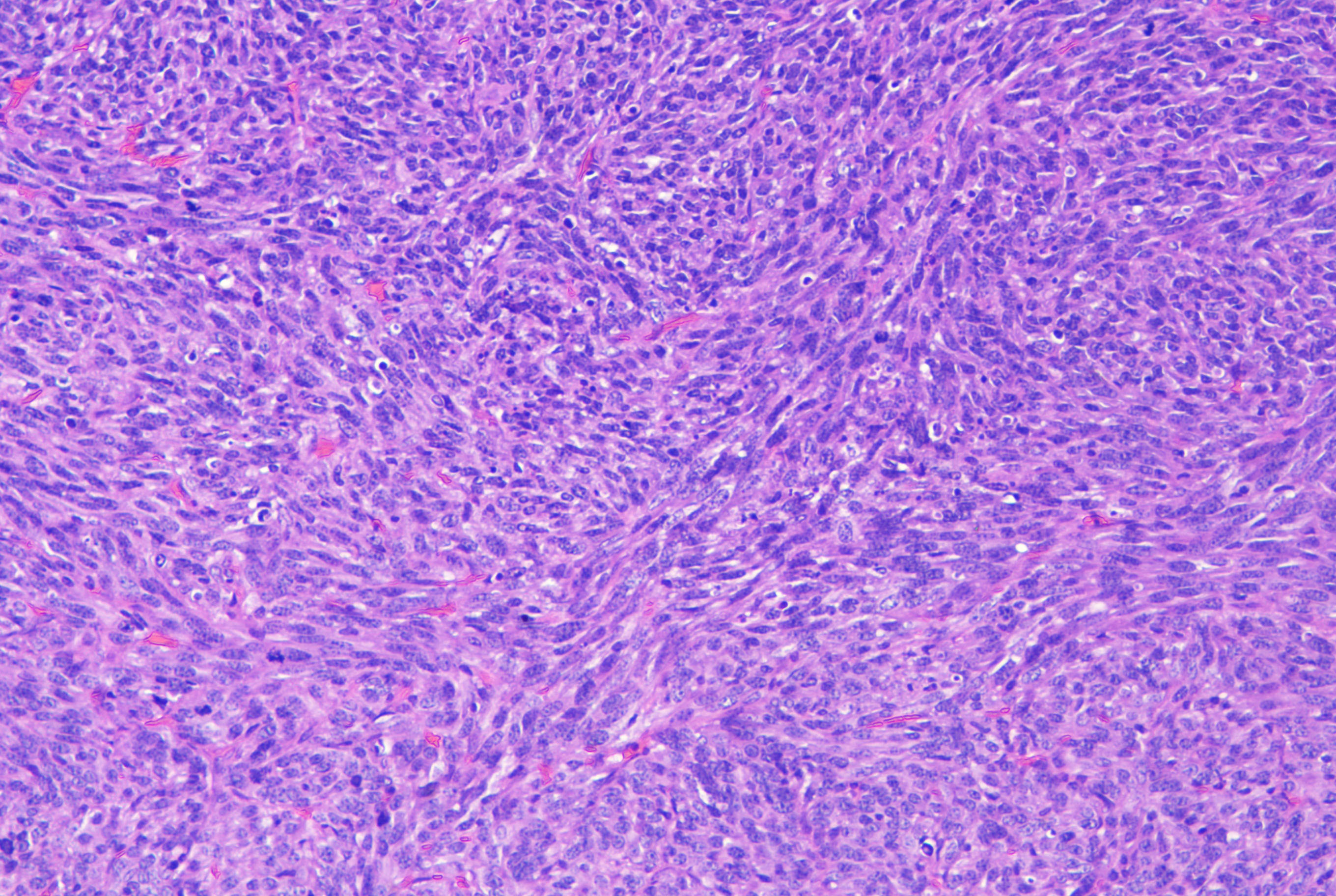

Supplement: Supplementary file 1 [file DataSheet1.zip › data/experiment/animal/DMSO 200X.jpg]

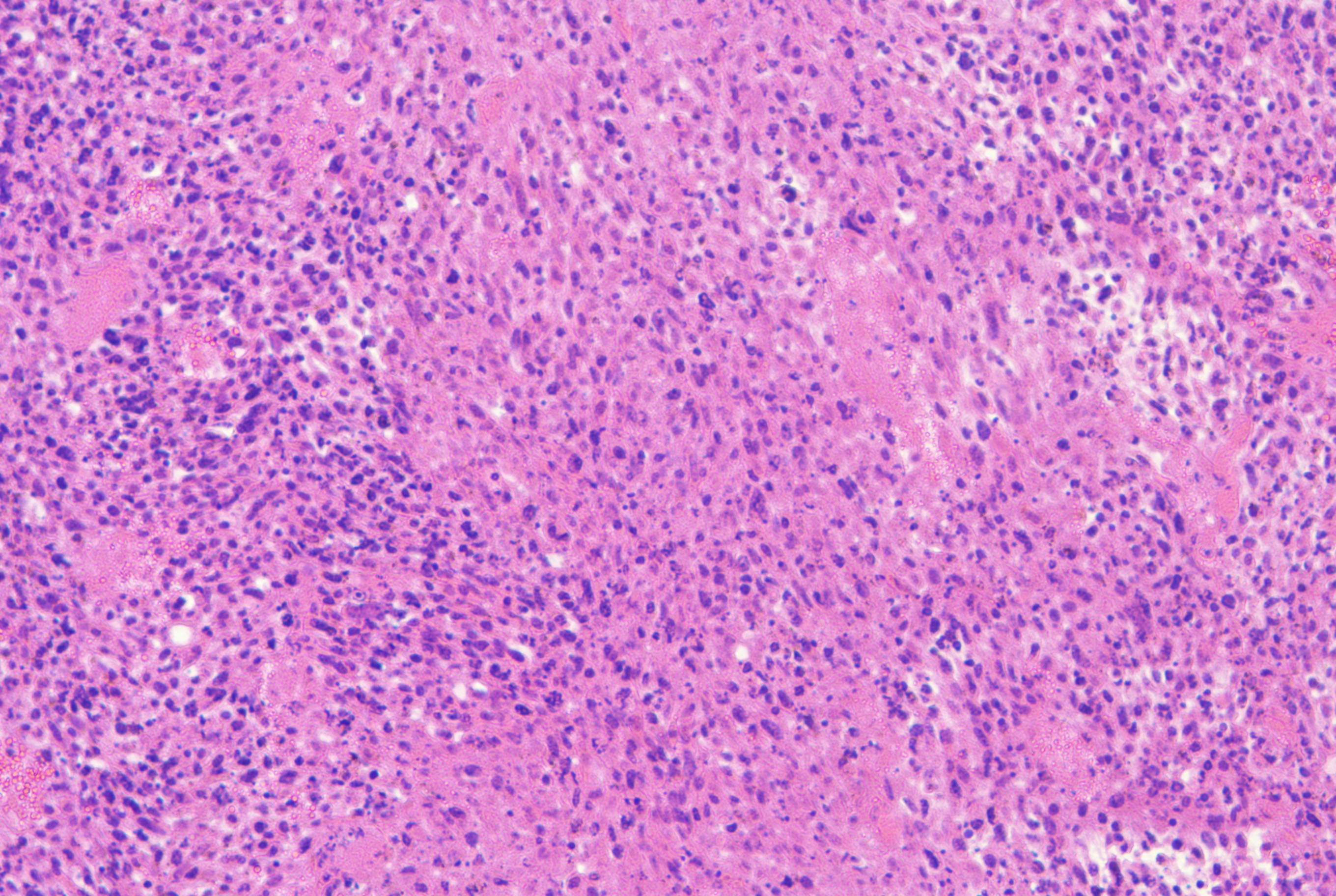

Supplement: Supplementary file 1 [file DataSheet1.zip › data/experiment/animal/HTS07545 200X.jpg]

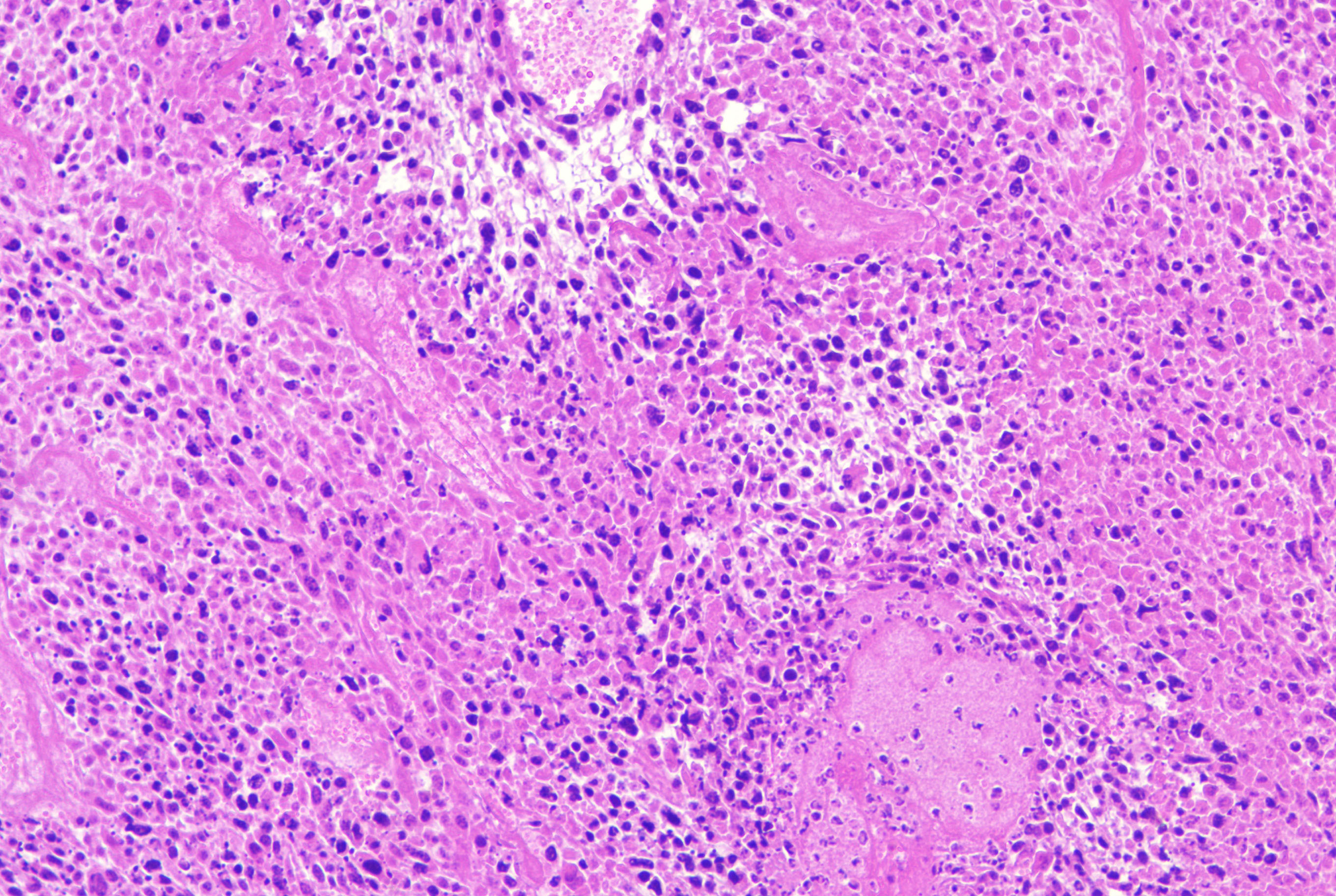

Supplement: Supplementary file 1 [file DataSheet1.zip › data/experiment/animal/Erastin 200X.jpg]
