# Supplementary material for: Comprehensive analysis of SQOR involvement in ferroptosis resistance of pancreatic ductal adenocarcinoma in hypoxic environments
Source: Front Immunol. 2025 May 1;16:1513589. doi: 10.3389/fimmu.2025.1513589 (PMC12078260; doi:10.3389/fimmu.2025.1513589)
Supplement: Supplementary file 2 [file DataSheet2.docx]

***Supplementary Material***

1. **Supplementary Figures**

**
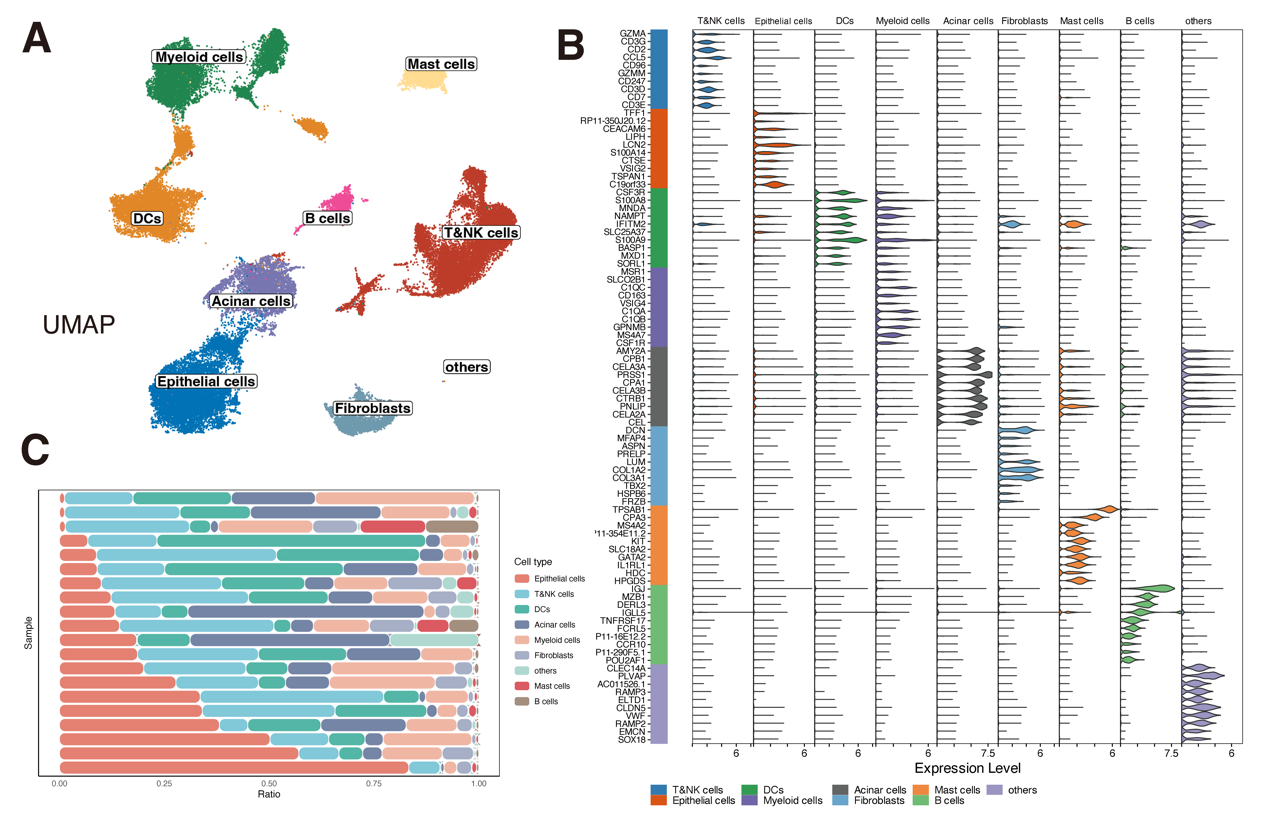
**

**Supplementary Figure S1 Single-cell annotations. A-B.** Single-cell dimensionality reduction, clustering, and annotation results. **C.** Cell scale diagram.

**
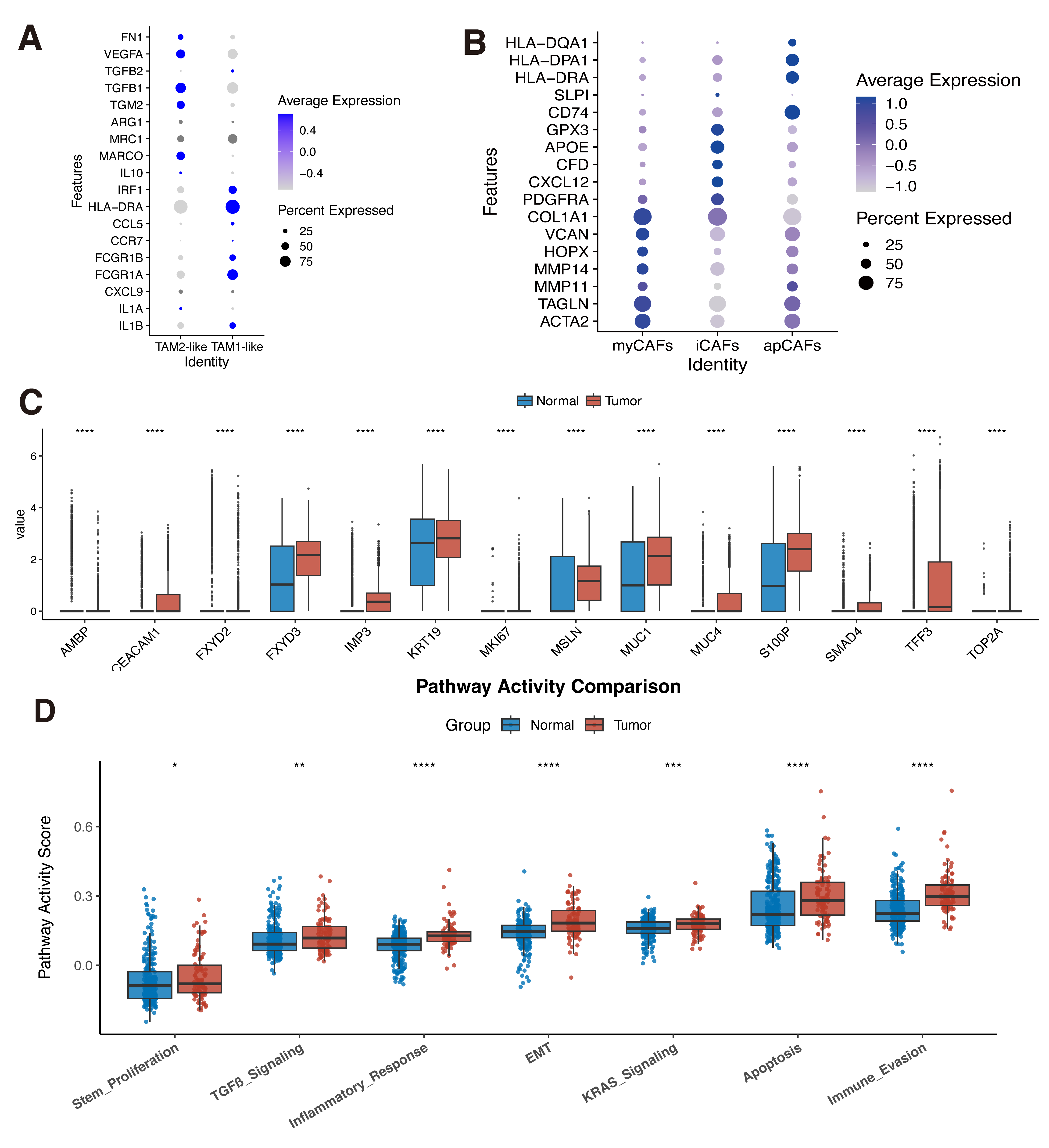
**

**Supplementary Figure S2 Single-cell specific cell type annotations. A.** Dotplot of marker genes for TAM1-like and TAM2-like. **B.** Dotplot of marker genes for myCAFs, iCAFs and apCAFs. **C.** Boxplot of marker genes for Pancreatic normal epithelial cells and tumour epithelial cells. **D.** Tumor-specific pathway score for Pancreatic normal epithelial cells and tumour epithelial cells. (Wilcoxon rank sum test). ns, P≥ 0.05; *, P<0.05; **, P<0.01; ***, P<0.001; ****, P<0.0001. TAM, tumour-associated macrophages; apCAFs, antigen-presenting fibroblasts; iCAFs, inflammatory fibroblasts; myCAFs, myofibroblasts.

**
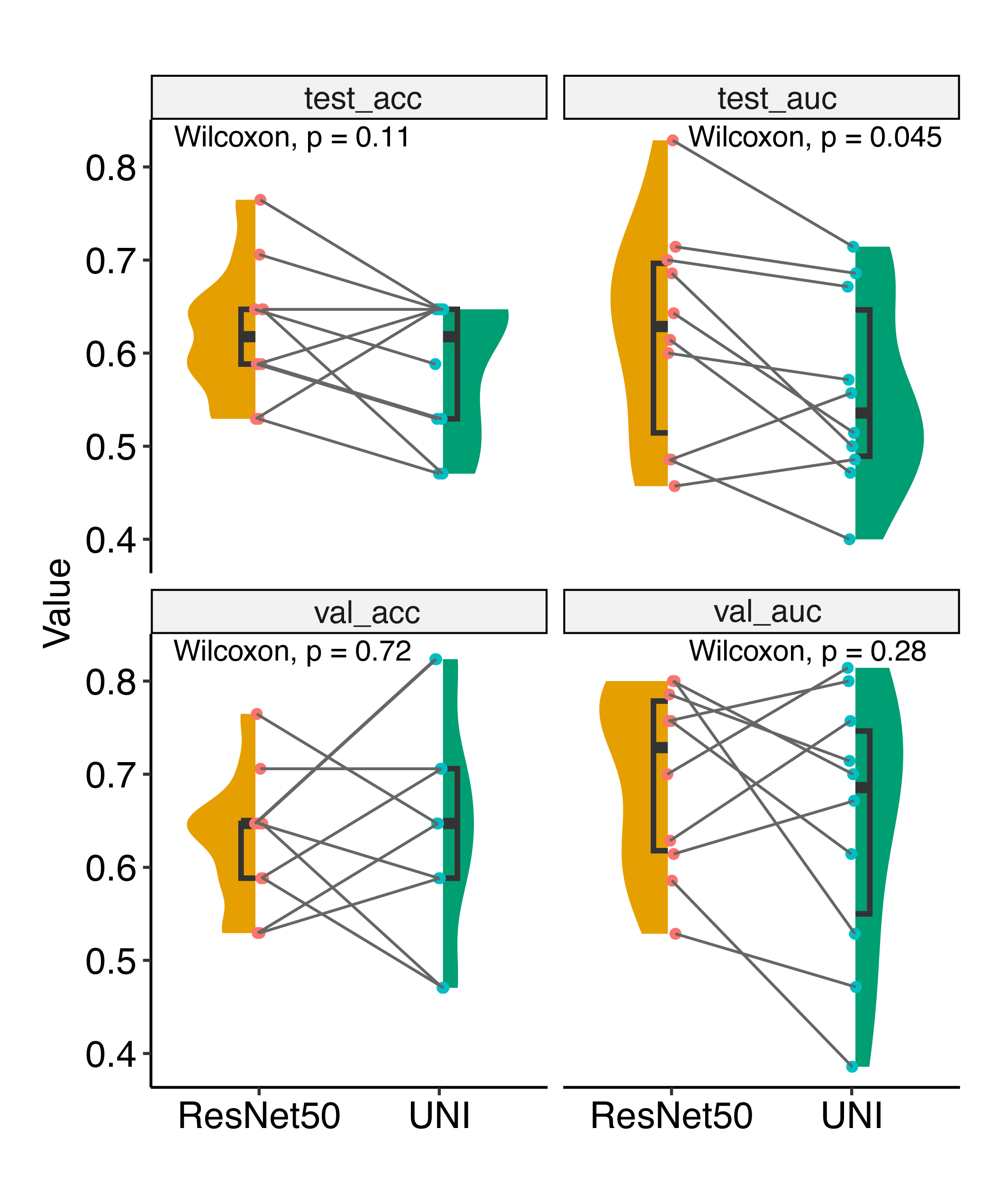
**

**Supplementary Figure S3 Performance comparison of ResNet50 and UNI pre-trained models on test sets and validation sets (Wilcoxon rank sum test).**

**
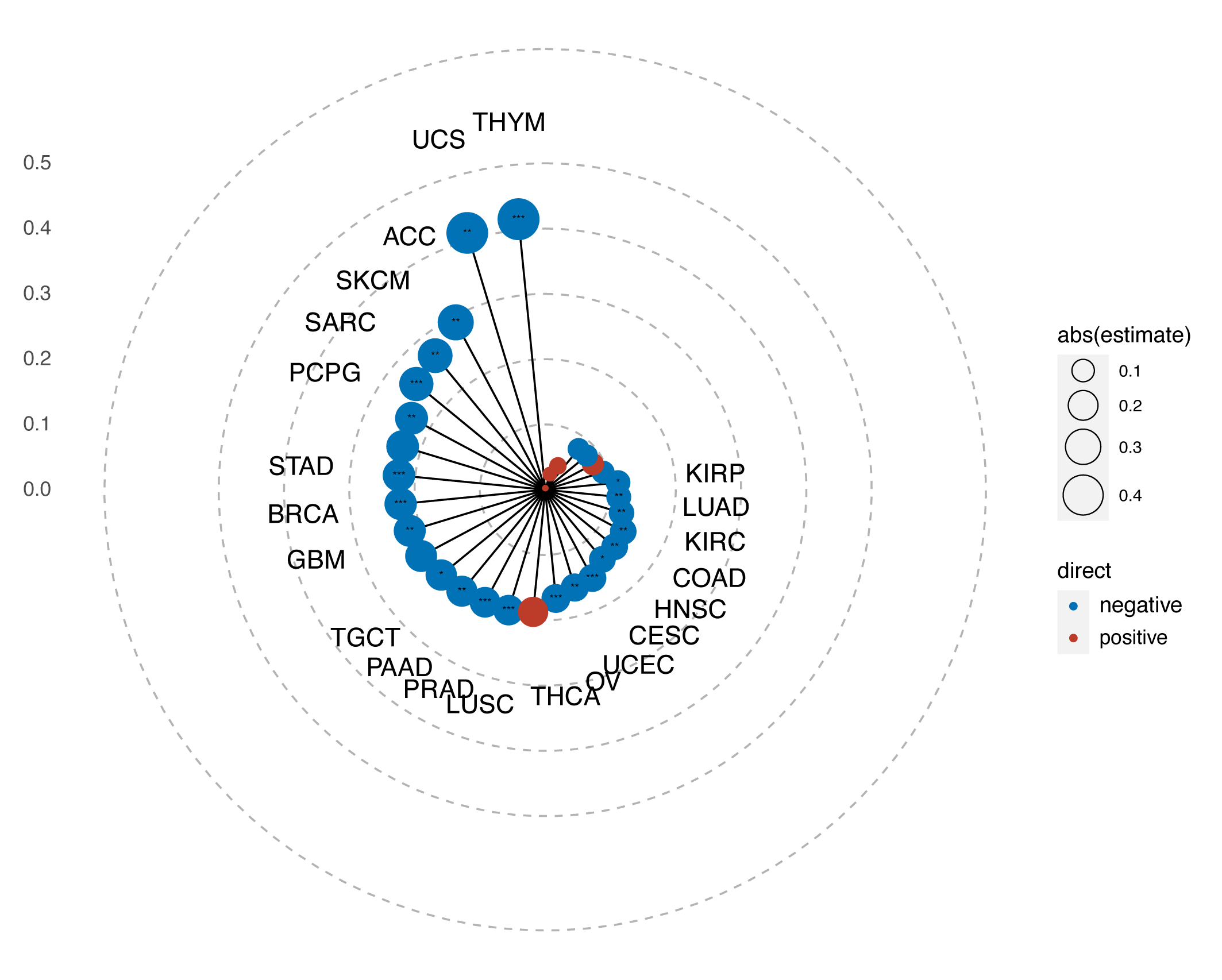
**

**Supplementary Figure S4 Correlation analysis of hypoxia and CD8^+^ T cells in the bulk dataset at the pan-cancer level (Spearman correlation).** KIRP, Kidney renal papillary cell carcinoma; LUAD, Lung adenocarcinoma; KIRC, Kidney renal clear cell carcinoma; COAD, Colon adenocarcinoma; HNSC, Head and Neck squamous cell carcinoma; CESC, Cervical squamous cell carcinoma and endocervical adenocarcinoma; UCEC, Uterine Corpus Endometrial Carcinoma; OV, Ovarian serous cystadenocarcinoma; THCA, Thyroid carcinoma; LUSC, Lung squamous cell carcinoma; PRAD, Prostate adenocarcinoma; PAAD, Pancreatic adenocarcinoma; TGCT, Testicular Germ Cell Tumours; GBM, Glioblastoma multiforme; BRCA, Breast invasive carcinoma; STAD, Stomach adenocarcinoma; PCPG, Pheochromocytoma and Paraganglioma; SARC, Sarcoma; SKCM, Skin Cutaneous Melanoma; UCS, Uterine Carcinosarcoma; THYM, Thymoma.


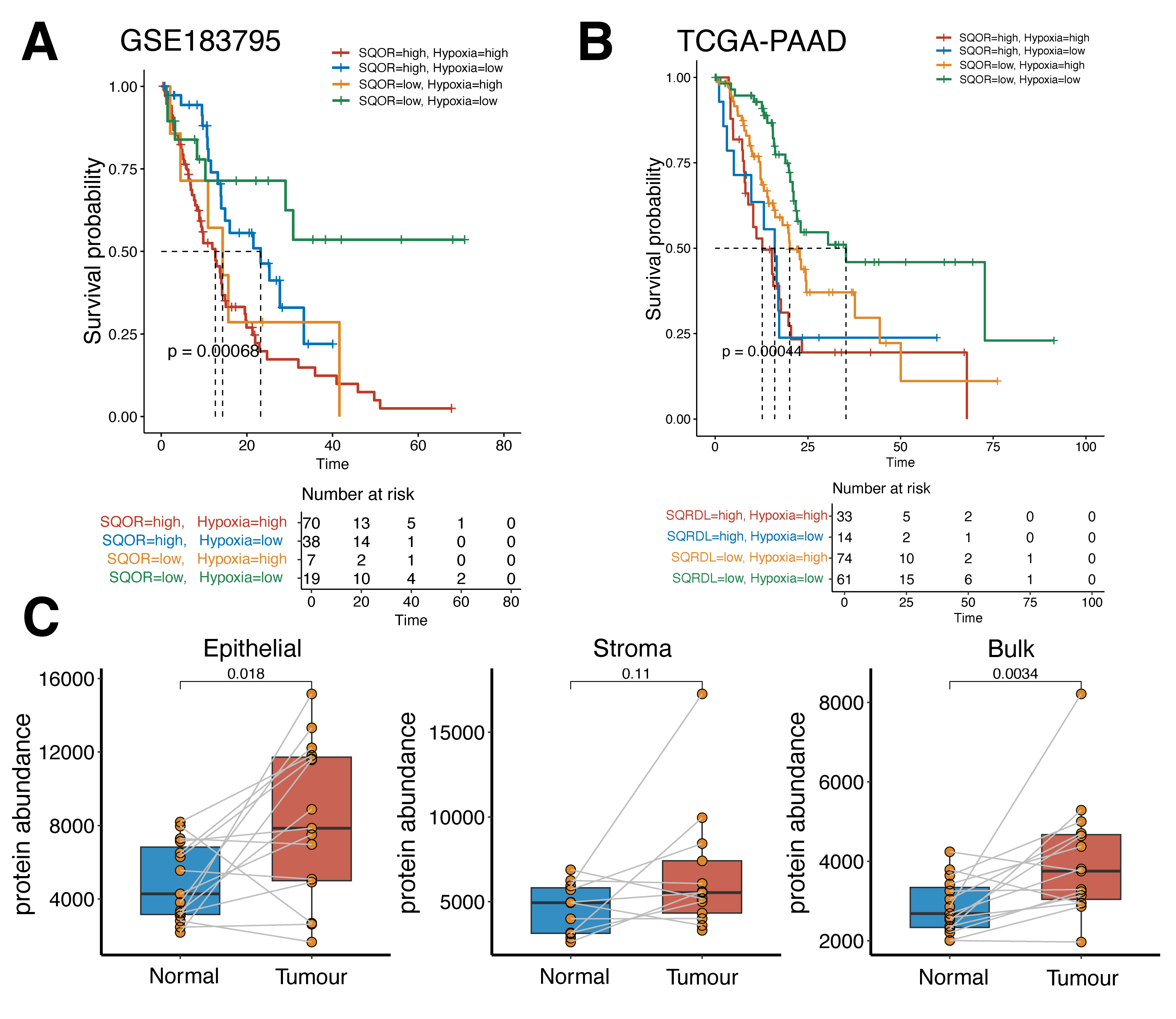


**Supplementary Figure S5 SQOR prognosis and analysis of differences. A.** Post-risk stratified KM survival analysis after combined hypoxia and SQOR in the GSE183795 dataset (Log-rank test). **B.** Post-risk stratified KM survival analysis after combined hypoxia and SQOR in the TCGA-PAAD dataset (Log-rank test). **C.** Proteomic distribution analysis of SQOR in tumours and normal tissues (Wilcoxon rank sum test). PAAD, Pancreatic adenocarcinoma; KM, Kaplan-Meier.

**
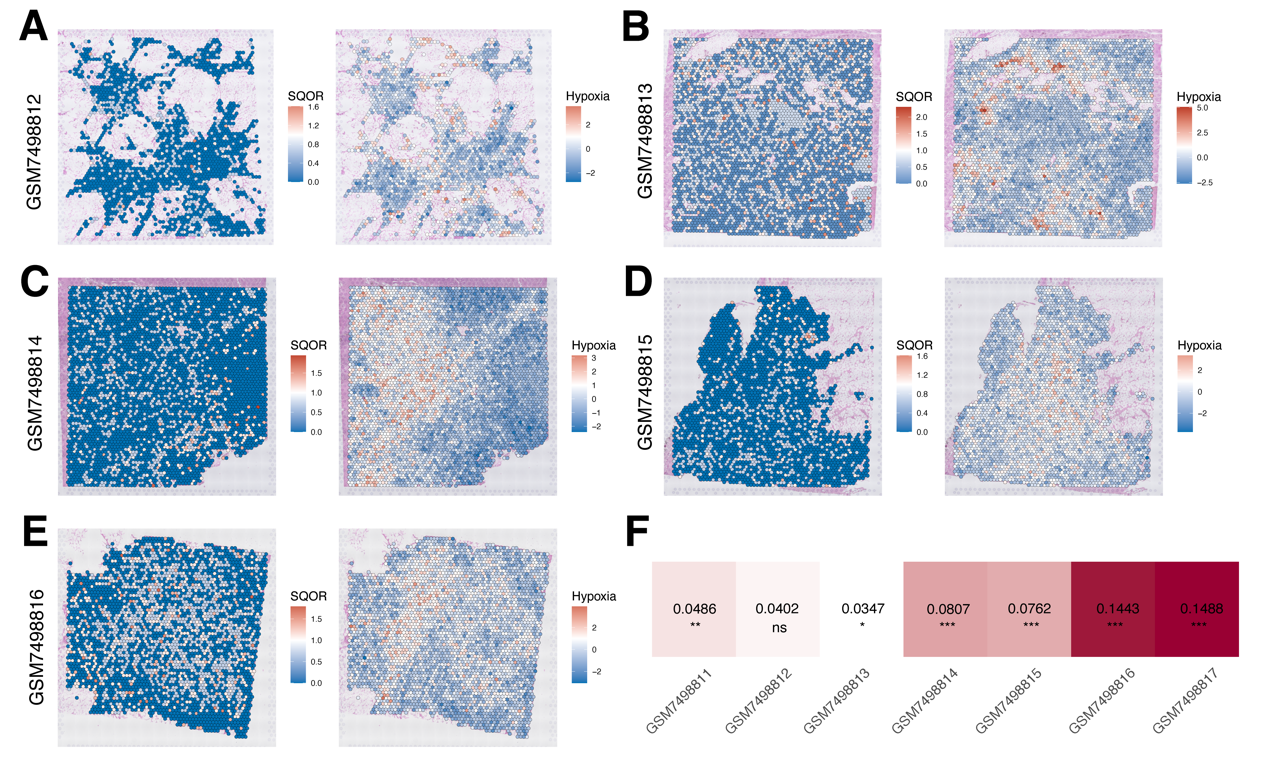
**

**Supplementary Figure S6 Spatial co-localisation of hypoxia and SQOR expression. A-E** Spatial localisation of SQOR and hypoxia. (A), sample GSM749812 (B), sample GSM749813 (C), sample GSM749814 (D), sample GSM749815 (E), sample GSM749816. **F.** Pan-cellular correlation analysis of SQOR and hypoxia in the spatial transcriptome (Spearman correlation).

**
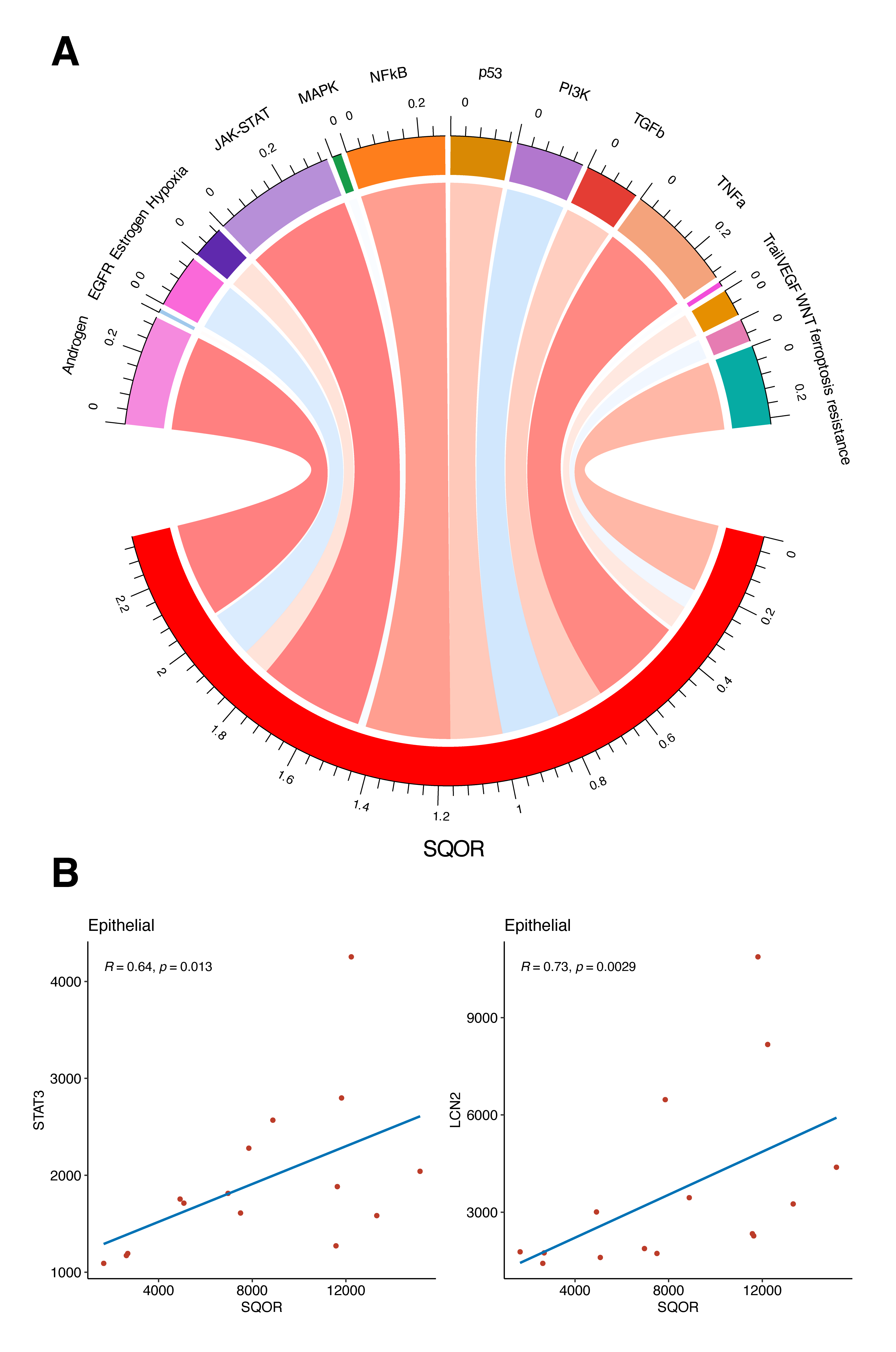
**

**Supplementary Figure S7 Correlation analysis of SQOR and multiple pathways.**

**A.** Correlation analysis of SQOR with multiple pathways/genes set scoring including hypoxia and ferroptosis resistance in single cell dataset at pan-cancer level (Spearman correlation). **B.** Correlation analysis of SQOR and ferroptosis resistance-related proteins in proteomics (Spearman correlation).

**
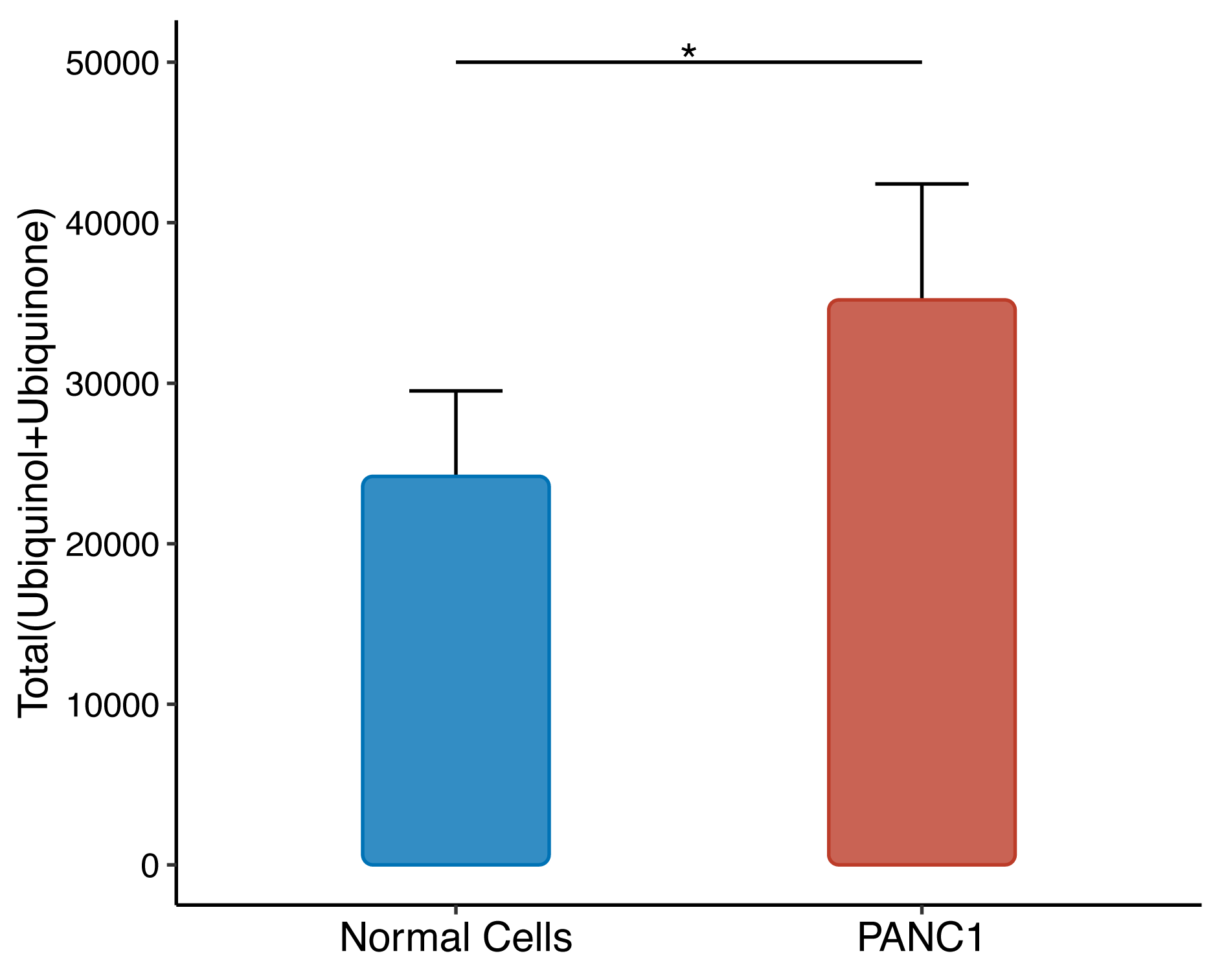
**

**Supplementary Figure S8 Differences in ubiquinone/ubiquinol distribution in pancreatic normal cells and PANC1 cells (Wilcoxon rank sum test).** *, P<0.05

1. **Supplementary Tables**

| **Public dataset sources** | | | |
| --- | --- | --- | --- |
| dataset | | sample | references |
| TCGA | | n=11057 | https://gdc.cancer.gov/about-data/publications/pancanatlas |
| GTEx | | n=5172 | https://gdc.cancer.gov/about-data/publications/pancanatlas |
| GEO | GSE183795 | n=134 | PMID: 36426859 |
|  | GSE155698 | n=20 | PMID: 36411320, 34296197 |
|  | GSE235315 | n=7 | PMID: 38297291 |

**Supplementary Table S1 Public dataset sources.**

| **Clinical Patient Baseline Data Sheet** | |
| --- | --- |
| Variable | Total（n=24） |
| **Age（y）** | 67.38±9.25 |
| **Sex（Male/Female）** |  |
| Male | 17(70.83%) |
| Female | 7(29.17%) |
| **pT** |  |
| T1 | 3(12.5%) |
| T2 | 11(45.83%) |
| T3 | 8(33.33%) |
| T4 | 2(8.33%) |
| **pN** |  |
| N0 | 16(66.67%) |
| N1 | 6(25%) |
| N2 | 2(8.33%) |
| **pTNM stage** |  |
| I | 11(45.83%) |
| II | 9(37.5%) |
| III | 4(16.67%) |
| **Maximum diameter (cm)** | 3.33±0.97 |
| **Normal tissues** |  |
| High expression | 12(50%) |
| Low expression | 12(50%) |
| **Tumour tissues** |  |
| High expression | 23(95.83%) |
| Low expression | 1(4.17%) |
|  |  |

**Supplementary Table S2 Clinical Patient Baseline Data Sheet.**

| **Marker genes for cell annotation** | | |
| --- | --- | --- |
|  | Marker gene | references |
| macrophage | C1QA, C1QB, C1QC, SELENOP, RNASE1, DAB2, LGMN, PLTP, MAF, SLCO2B1 | PMID: 34035069 |
| monocyte | VCAN, FCN1, CD300E, S100A12, EREG, APOBEC3A, STXBP2, ASGR1, CCR2, NRG1 | PMID: 34035069 |
| TAM1-like | IL1B, IL1A, CXCL9, FCGR1A, FCGR1B, CCR7, CCL5, HLA-DRA, IRF1 | PMID: 33144684 |
| TAM2-like | IL10, MARCO, MRC1, ARG1, TGM2, TGFB1, TGFB2, VEGFA, FN1 | PMID: 33144684 |
| myCAF | ACTA2, TAGLN, MMP11, MMP14, HOPX, VCAN, COL1A1 | PMID: 31197017, 33930309 |
| iCAF | PDGFRA, CXCL12, CFD, APOE, GPX3 | PMID: 31197017, 33930309 |
| apCAF | CD74, SLPI, HLA-DRA, HLA-DPA1, HLA-DQA1, COL1A1 | PMID: 31197017, 33930309 |
| tumour | S100P, MUC1, MSLN, SMAD4, CEACAM1, MUC4, KRT19, IMP3, AMBP, FXYD2, FXYD3, TOP2A, MKI67, TFF3 | PMID: 34326612, 29115542, 27689616, 16003754 |

**Supplementary Table S3 Marker genes for cell annotation.**

| **Ferroptosis Suppressor** |
| --- |
| GPX4 |
| NFE2L2 |
| SLC7A11 |
| FTH1 |
| SCD |
| CBS |
| FTMT |
| MTOR |
| AIFM2 |
| CDH1 |
| FXN |
| HMOX1 |
| HSF1 |
| HSPA5 |
| LCN2 |
| PLA2G6 |
| SLC40A1 |
| SQSTM1 |
| STAT3 |
| AKR1C1 |
| AKR1C2 |
| AKR1C3 |
| ATF4 |
| NQO1 |

**Supplementary Table S4 Ferroptosis suppressor.**

| **Log-rank test after risk stratification of hypoxia and SQOR risk** | | | |
| --- | --- | --- | --- |
|  | | | |
| TCGA-PAAD | | | |
|  | high-hypoxia high-SQOR | high-hypoxia low-SQOR | low-hypoxia high-SQOR |
| high-hypoxia low-SQOR | 0.07088 | - | - |
| low-hypoxia high-SQOR | 0.93367 | 0.29406 | - |
| low-hypoxia low-SQOR | 0.00017 | 0.04212 | 0.01109 |
|  | | | |
| GSE183795 | | | |
|  | high-hypoxia high-SQOR | high-hypoxia low-SQOR | low-hypoxia high-SQOR |
| high-hypoxia low-SQOR | 0.7469 | - | - |
| low-hypoxia low-SQOR | 0.0201 | 0.532 | - |
| low-hypoxia low-SQOR | 0.0046 | 0.1335 | 0.2841 |

**Supplementary Table S5 Log-rank test after risk stratification of hypoxia and SQOR risk.**

| **Results of multifactorial cox regression analysis in pancreatic ductal adenocarcinomas** | | | |
| --- | --- | --- | --- |
| factors | coef | HR | Pvalue |
| SQOR | 0.485 | 1.624(1.102-2.392) | 0.014 |
| age | 0.02 | 1.02(0.999-1.042) | 0.065 |
| tumor_dimension | 0.048 | 1.049(0.914-1.204) | 0.497 |
| histologic_grade | 0.109 | 1.115(0.811-1.533) | 0.502 |
| pathologic_N | 0.624 | 1.866(1.073-3.247) | 0.027 |
| pathologic_T | 0.285 | 1.33(0.672-2.631) | 0.413 |
| gendermale | -0.129 | 0.879(0.564-1.371) | 0.571 |
| stage | -0.228 | 0.796(0.376-1.685) | 0.551 |

**Supplementary Table S6 Results of multifactorial cox regression analysis in pancreatic ductal adenocarcinomas.**
